# Da Vinci Project: Educating Sustainability Change-Makers with Transdisciplinary Challenge-Based Learning and Design Thinking

**DOI:** 10.1021/acs.jchemed.4c00158

**Published:** 2024-09-17

**Authors:** Fieke Sluijs, Sabine G. Uijl, Eelco T.C. Vogt, Bert M. Weckhuysen

**Affiliations:** † Inorganic Chemistry and Catalysis Group, Institute for Sustainable and Circular Chemistry (ISCC), Department of Chemistry, Faculty of Science, Utrecht University, Universiteitsweg 99, 3584 CG Utrecht, The Netherlands; ‡ Alliance of TU/e, WUR, UU, and UMC Utrecht (EWUU), Princetonlaan 6, 3584 CB Utrecht, The Netherlands

**Keywords:** upper-division undergraduate, interdisciplinary/multidisciplinary, problem-solving/decision-making, learning theories, challenge-based learning, design thinking

## Abstract

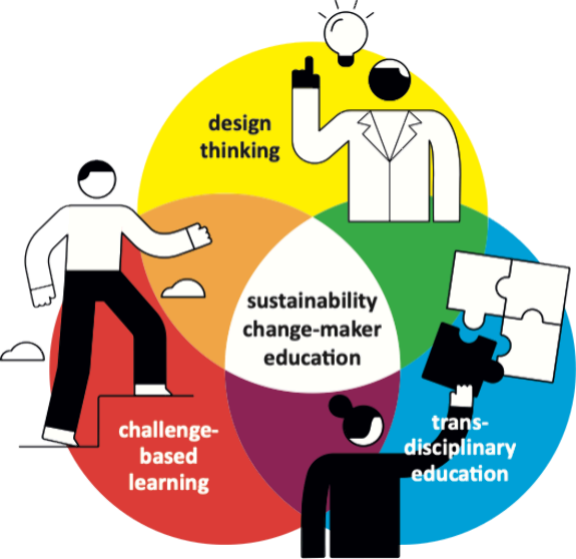

Sustainability transitions need professionals with specific skills and attitudes that students often do not develop in their regular chemistry education. To foster sustainability change-maker competencies, we suggest augmenting higher education curricula, e.g., chemical degree programs, with transdisciplinary challenge-based learning combined with design thinking. The Da Vinci Project at Utrecht University (UU) in The Netherlands explores this approach, aiming to cultivate the undergraduates’ sustainability change-maker competencies. After five years of experience, we reflected on the students’ learning outcomes in this UU honors program. We conclude that transdisciplinary challenge-based education combined with design thinking provides unique opportunities for students to develop valuable skills and attitudes for navigating sustainability transitions, including the transition toward sustainable chemistry. These involve collaboration, communication, creative thinking, integrative problem-solving, stakeholder engagement, openness, empathy, the ability to deal with uncertainty and complexity, self-awareness, critical reflection, courage, and perseverance.

## Introduction

Few other masterminds in history were as creative in so many different areas as Leonardo da Vinci. He painted the Mona Lisa, cut open a still beating heart of a slaughtered pig to discover how the heart valves worked, explained the reflection of Earth’s light on the moon, invented hydraulic pumps, and designed and played musical instruments, one of which was inspired by the mechanism of the larynx,^1,2^ all without any formal education.^3^ Yet, we can learn countless lessons from this epitome of *uomo universalis*. His work illustrates the potency arising from the intersection of art, science, and technology, showcasing the effectiveness of interdisciplinarity.^4^ If we learn to regain our childlike wonder, attentively observe, connect our imagination to our intellect, transcend disciplines, and experiment to learn, we can “change” the world around us. This is what the Da Vinci Project at Utrecht University (UU, The Netherlands) aims at, to empower undergraduates to drive fundamental change.^5^

Chemistry can play a significant role in finding solutions to complex sustainability problems if we better prepare future chemists for that role.^6^ This requires (1) better understanding of the competencies required to address sustainability challenges and (2) new teaching and learning approaches that enable chemistry students to develop such abilities. The transition toward a more sustainable future implies a transformation toward sustainable and circular chemistry,^7^ for which chemists are forced to cross disciplinary boundaries to understand problems from a holistic perspective and to successfully arrive at integrative solutions.^8,9^ The entire lifecycle of chemical products and the related system of actors, institutions and culture must be considered.^7^ However, chemical education has been largely reductionist, an approach which is not fully adequate to tackle sustainability challenges.^10^ Therefore, many educators begin to include systems thinking in chemistry curricula,^10−13^ and researchers recently show great progression in developing scaffolding and assessment instruments regarding systems thinking skills.^14,15^ However, while systems thinking is crucial for a holistic understanding of complex problems,^16^ it is not enough to equip chemists with all competencies associated with sustainability change-makers. Talanquer et al.^17^ propose a competency framework with systems thinking as the underpinning form of reasoning, but they also identify social-environmental responsibility and collaborative problem-solving as core competencies. We think these competencies imply strong inter- and intrapersonal skills, such as empathy, openness, and self-awareness, as well as creativity and integrative skills. Moreover, according to Talanquer et al.,^17^ future chemists should not only be able to understand complex sustainability problems, but also to manage complexity, change, uncertainty, resilience, and vulnerability to develop sustainable solutions.^17^ Systems thinking competence does not cover all of these abilities.

Higher chemical education is still mainly knowledge-driven, teacher-centered, and instruction-based, an approach that has been proven insufficient to foster important sustainability competencies, such as social-environmental responsibility and collaborative problem-solving.^14,17^ We believe that transdisciplinary education, challenge-based learning (CBL), and design thinking can serve as useful pedagogies for chemistry students to develop skills and attitudes associated with sustainability change-makers. Much has been published on the potential of, respectively, transdisciplinary education, CBL, and design thinking to develop sustainability competencies, but a scientific knowledge gap exists on the effects of a combination of these approaches in academic education. Transdisciplinary education and CBL stimulates crucial skills for sustainability problem-solving, such as adaptability, collaboration, communication, critical thinking, and leadership.^6,18^ By engaging students in resolving real-life open-end challenges that exist in their environments,^19^ CBL facilitates self-directed learning, nurturing T-shaped professionals.^18^ Design thinking is a useful approach to develop solutions for complex problems,^20^ and it has much potential to facilitate successful sustainability-oriented innovation.^21^ Experiencing the design process fosters valuable attitudes, such as empathy, curiosity, confidence, resilience, and adaptability.^22^ Though a combination of transdisciplinary CBL and design thinking seems obvious, there is a lack of evidence of the impact this combination has on the learning outcomes for students.

This Article shows the results of educational design research on the Da Vinci Project, a transdisciplinary CBL experience with design thinking as a methodology for integrative problem-solving. It can serve as an example for curriculum designers with the ambition to educate sustainability change-makers. The research objective was to discover if and how transdisciplinary CBL in combination with design thinking fosters the students’ skills and attitudes associated with sustainability change-maker competencies. While open-end learning is an essential characteristic of CBL, learning outcomes are not strictly determined beforehand.^18,23^ General learning objectives were predefined in the Da Vinci Project to assess the students’ progress and deliverables, but students also pursued personal learning objectives. Hence, we assumed the actual learning outcomes reach beyond the predefined objectives. Qualitative research was conducted to identify self-perceived learning outcomes, to benchmark the outcomes with existing sustainability competency frameworks, and to draw conclusions on which aspects of the learning experience stimulate change-maker competencies.

### Da Vinci Project

The Da Vinci Project is set up as a university-wide honors program at Utrecht University (UU), open to Bachelor’s degree students from all faculties, ranging from Science, Medicine, Veterinary Medicine, and Geosciences to Humanities, Law, Economics and Governance, and Social and Behavioral Sciences. The project was initiated to let chemistry students get acquainted with the transdisciplinary character of chemistry-related sustainability challenges and to invite students with other backgrounds to gain insights into their potential role. The initiators wanted to facilitate students to learn to (1) collaborate in a transdisciplinary and multistakeholder context, (2) navigate in the uncertainty and complexity of the problem-solution space of sustainability challenges, and (3) develop creative confidence and resilience to drive innovation. From 2019 to 2024, the 20-week program took place yearly, and 113 students from more than 30 different Bachelor’s degree courses participated, of which four were chemistry students.

In the project, students work in interdisciplinary teams of four to six students, tackling sustainability challenges in collaboration with societal partners. One team worked on the challenge of making circular paint feasible in collaboration with a paint manufacturer. Another team collaborated with an NGO that cleans oceans and rivers from plastic waste to separate organic material from plastics. Other challenges were, for example, to reduce the waste of chemicals in a research lab, how to turn a new chemical technology of converting CO_2_ to formic acid into a business case, and how contaminated gloves could be reused or recycled. On davinciproject.nl, more information about the project, the challenges, and the partners can be found.

### Sustainability Change-Maker Competencies

**Figure 1:**
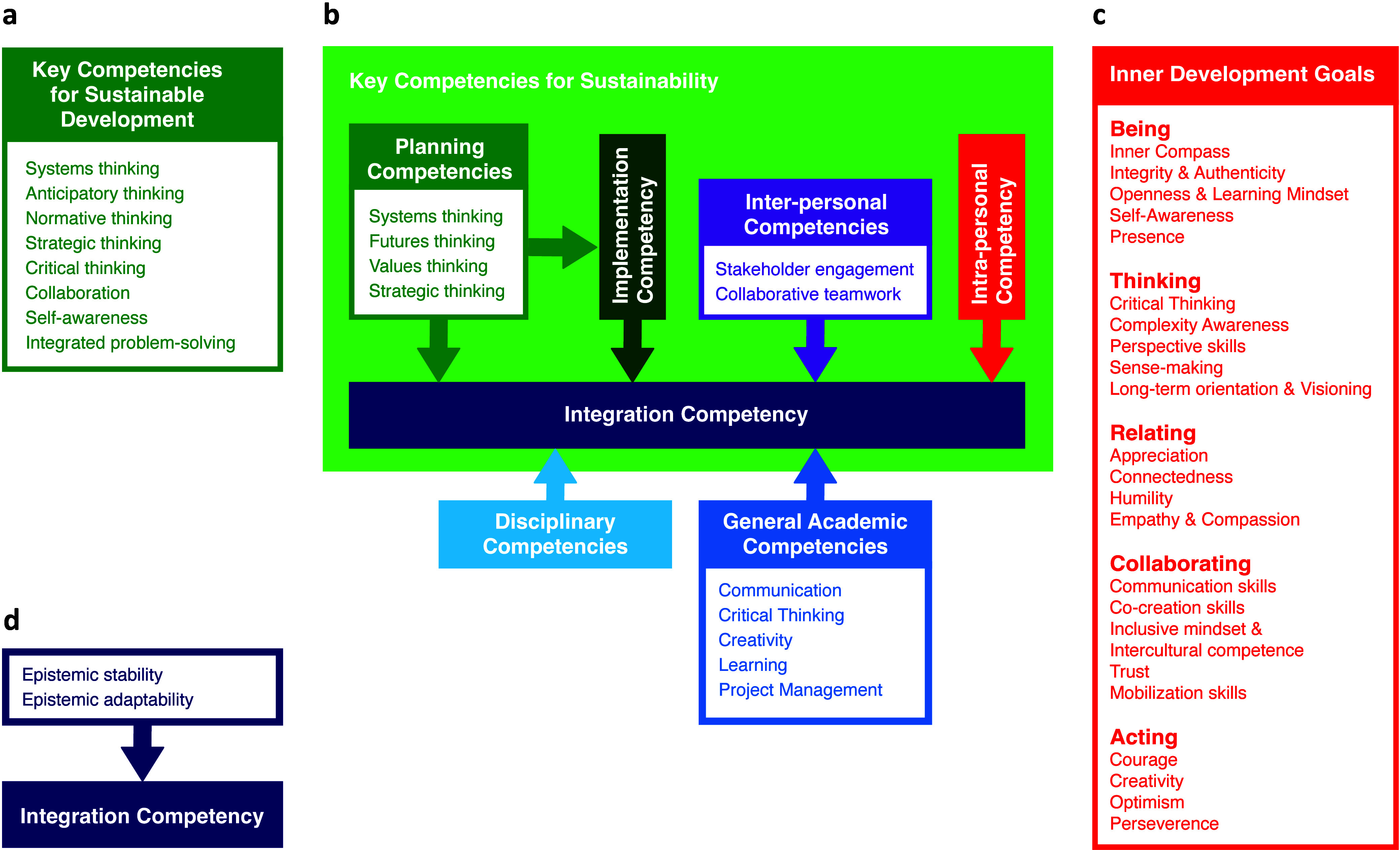
Models of sustainability change-maker competencies used to benchmark the learning outcomes of the Da Vinci Project. (a) List of key competencies for sustainable development by UNESCO.^24^ (b) An adapted and modified version of the frameworks of sustainability competencies presented by Brundiers et al.^26^ and Wiek and Redman.^27^ The lighter green area presents the key competencies for sustainability as Brundiers et al.^26^ and Wiek and Redman^27^ propose. These key competencies are further defined in capacities in connected white blocks. All arrows point at the integration competency, the ability to combine and integrate all other key competencies, as well as disciplinary and general academic competencies for sustainability problem-solving. (c) The Inner Development Goals (IDGs) framework,^28^ consisting of five dimensions and 23 transformational qualities for sustainable development. These partly overlap with key competencies for sustainability and general academic competencies in the framework of Brundiers et al.^26^ and Wiek and Redman.^27^ (d) The conditional relationship of epistemic stability and epistemic adaptability with the integration competency based on the theory of interdisciplinary knowledge integration by Horn et al.^30^

To benchmark the learning outcomes in the Da Vinci Project, we used various theoretical frameworks of sustainability change-maker competencies, as presented in Figure 1. The concept “sustainability change-maker” originates from UNESCO’s report on education for the SDGs.^24^ The report provides eight key competencies for sustainable development presented in Figure 1a. A key competency for sustainable development can be defined as a functionally linked complex of knowledge, skills, and attitudes facilitating effective task performance and problem-solving in real-world sustainability contexts.^25^ The key competencies for sustainability have been a popular topic before and after UNESCO’s report, resulting in a widely accepted framework created by Brundiers et al.^26^ and Wiek and Redman,^27^ presented in Figure 1b.

The Inner Development Goals (IDGs) were also used for our benchmark, a framework developed by a global community dissatisfied with SDG progress,^28,29^ shown in Figure 1c. Comprising five dimensions (being, thinking, relating, collaborating, and action) and 23 transformational skills and qualities for sustainable development, the IDGs aim to address humanity’s struggle with complexity hindering SDG achievement.^28,29^

Also included in the benchmark is the theory of interdisciplinary knowledge integration by Horn et al.,^30^ which will be explained in the following paragraph. An overview of the key competencies for sustainability, the IDGs, and their definitions is available in the Supporting Information.

Except for UNESCO’s list, the models in Figure 1 were not yet published when the Da Vinci Project was designed. Hence, the learning outcomes the designers envisioned (available in the Supporting Information) are not articulated according to these frameworks. Moreover, the predefined learning outcomes were not articulated in terms of (key) competences. However, the researchers assume that the students develop (some of) these sustainability change-makers competencies.

### Transdisciplinary Education, an Interdisciplinary Approach

The term *transdisciplinary education* refers to the involvement of nonacademic stakeholders or as an entirely new discipline integrating and implementing science.^31^ The Da Vinci Project exemplifies the first interpretation. We use the term “*interdisciplinary*” when we refer to a specific approach. An *interdisciplinary approach* is integrating knowledge and methods from different disciplines, using a real synthesis of approaches, opposed to a *multidisciplinary* approach in which students work together without integrating knowledge and methods.^6^

Involving chemistry undergraduates in transdisciplinary education is highly desirable because chemistry connects many scientific disciplines in sustainability transitions.^6^ It equips chemistry students with vital skills for integrative sustainability problem-solving, enhancing their societal value. However, assembling students from diverse disciplines in a team with a challenge does not automatically lead to integrative solutions. According to Horn et al.,^30^ students only develop integration competency if they mastered epistemic stability and epistemic adaptability. Epistemic stability involves contributing one’s academic knowledge confidently, while adaptability requires curiosity, openness, and communication skills to engage with others’ academic knowledge. Integrative competencies encompass a pluralistic understanding of topics (allowing multiple equally valid interpretations to coexist) and functional disagreement (addressing misunderstanding, disagreement, and conflict, seeing them rather as learning opportunities). As epistemic stability and epistemic adaptability are conditional for integrated problem-solving, we included these abilities in our benchmark.

### Challenge-Based Learning (CBL)

Competencies for sustainable development cannot be taught, but must be developed by the learners themselves during action, through experience and reflection.^32^ CBL proves to be ideal to acquire sustainability competencies because it engages students in authentic challenges in collaboration with societal actors, facilitating learning through action, experience, and reflection.^18,23,33^ Meaningful learning occurs when students reflect on challenging experiences, triggered by the experiences themselves and by feedback.^34^ Typical characteristics of CBL are real-life open-end challenges, focus on global themes, self-directed learning, interdisciplinarity, and a shift from traditional instruction to a coaching role for academic teachers.^23^ The open-end character means there are multiple possible approaches and outcomes, allowing students to both discover problems and design solutions, therefore it is difficult to predeterminate learning outcomes and assessment is often process-focused.^18,23^ CBL is increasingly popular in higher education to align the acquisition of disciplinary knowledge with the development of transversal competencies, such as collaboration and innovation.^35^

### Design Thinking

Design thinking is a useful methodology with an extensive toolset to tackle complex problems and develop innovative solutions, including solutions for sustainability challenges.^20,21,36^ It is an analytic and creative approach that engages participants in opportunities to experiment, create and prototype models, gather feedback, and redesign.^37,38^ The concept of design thinking usually refers to human-centered design practice beyond the professional design context, when people without a design background iteratively develop innovative products, systems, and services.^39,40^

**Figure 2:**
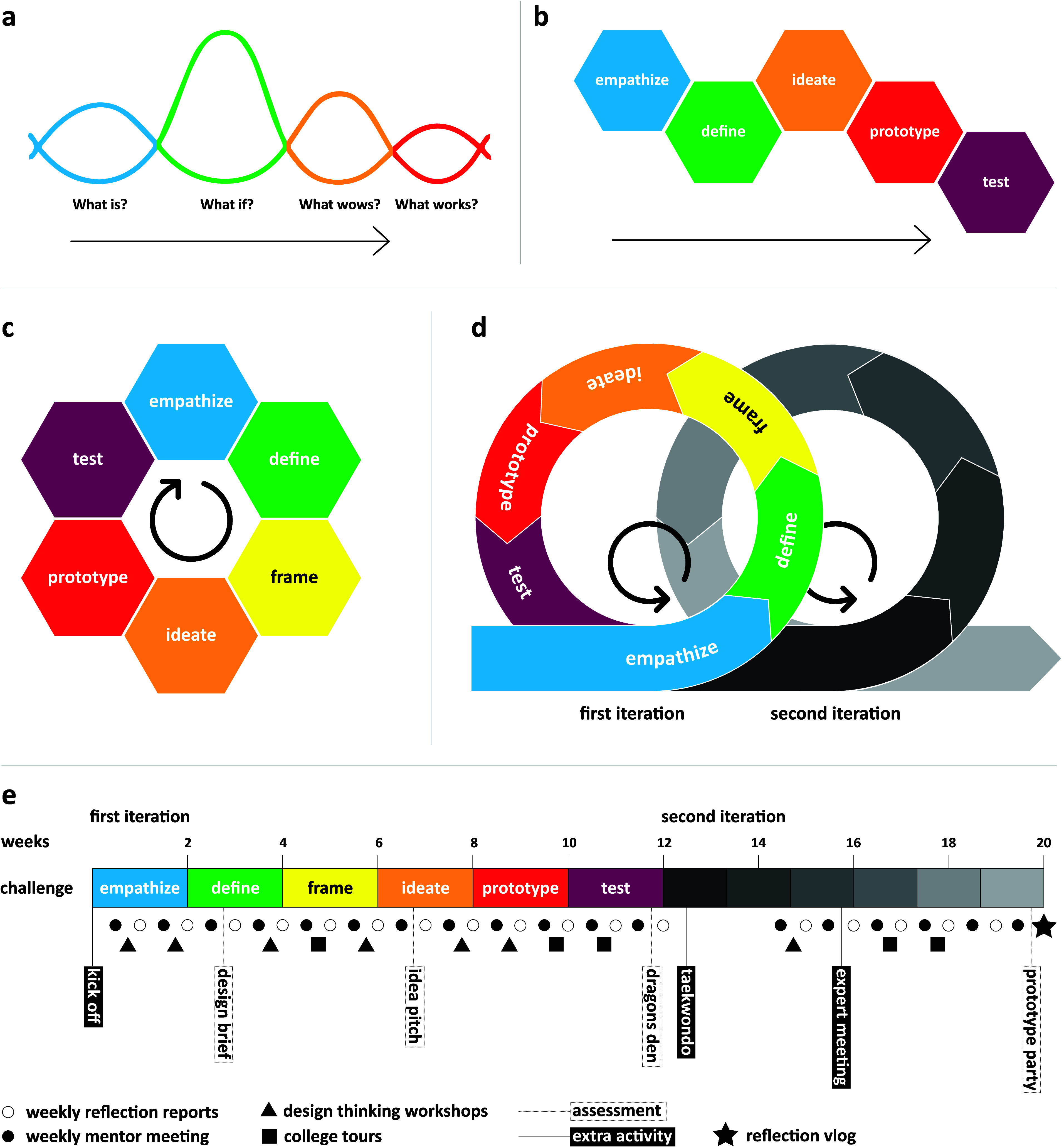
Evolution of design thinking models during the development of the Da Vinci Project. The first documents on the project’s program design refer to Liedtka’s four-question design thinking approach,^55^ shown in (a). In the pilot of the Da Vinci Project in 2019, the original model of the five-step process of the *Stanford d.School*(56) (b) is adapted and modified, shown in (c). The modified version better represents the iterative character of the process, and an additional step is added: *frame*. The pilot phase consisted of one full iteration. From the second until the last edition, the project included two iterations, as shown in (d). (e) Timeline of the program of the second to the last edition, including the design thinking stages, learning activities, and assessments.

The *Stanford d.School Model*(41) (Figure 2b), was adapted and applied in the Da Vinci Project. The model comprises five steps:

1. Empathize: conducting ethnographic research, immersion in world of end-user.
2. Define: interpreting data, defining the problem.
3. Ideate: generating ideas for problem-solving.
4. Prototype: making ideas tangible.
5. Test: validating ideas, testing prototypes.

While design thinking provides a useful framework to engage students in complex problem-solving,^37,42−44^ it also has pitfalls. With a framework like the *d.School Model*, students can structure an otherwise messy process but blindly following the steps is insufficient. People fail to successfully apply design thinking without the appropriate mindset.^45,46^ The reality of design thinking is much more chaotic, approaches and processes differ per challenge, and sustainability challenges are too complex to solve in a simple five-step procedure.^47^ Vignoli et al. define this mindset describing ten aspects: dealing with uncertainty and risk, empathy, holistic thinking, collaboration and diversity, learning oriented, experimentation, critical questioning, abduction, creative confidence, and optimism to create value.^46^ Therefore, when design thinking is applied in education, facilitating an environment in which students can develop this mindset is just as important as providing them with the method and tools.

A combination of CBL and design thinking is logical and has already been applied successfully in academic education before, for example, in engineering programs.^48−50^ CBL is relatively new, and design thinking can offer the stability it needs to evolve into an effective pedagogy tool.^51^ CBL requires an iterative process in which divergent and convergent reasoning are alternated to bring students from challenge to solutions,^18^ and design thinking provides a methodology and toolset for this iterative development.

## Program Design

The Da Vinci Project is designed according to the pedagogical principles of transdisciplinary education, CBL, and design thinking. Students work in interdisciplinary teams alongside societal partners to solve sustainability challenges. They are guided by various activities from briefing to a validated prototype, including coaching by a mentor, design thinking workshops, college tours, and presentations. Feedback and reflection are integral components of the program. In weekly reflection reports, students reflect on their personal development, and in team meetings they reflect on team performance. Students are also encouraged to actively seek feedback from peers, mentors, experts, and stakeholders, complemented by formal feedback events: an idea pitch, a dragon’s den, an expert meeting, and a prototype party.

A modified version of the *Stanford d.School model* is applied in the Da Vinci Project to structure the problem-solving process. While the original model (Figure 2b) provides a clear structure for students inexperienced with design, its linear representation contradicts the iterative nature of design thinking. Another additional weakness of the original model is the absence of a crucial design practice: *Framing*. Framing is the process of generating meaningful perspectives on the problem, which is characterized by abductive reasoning.^52^ This is critical to arrive at innovative solutions.^53,54^ The adapted model (Figure 2c) rectifies these issues by representing the iterative process and incorporating framing as a distinct stage. This modification allows interdisciplinary teams to deliberately explore a variety of viewpoints and create inspiring, integrative frames. The first edition of the Da Vinci Project involved one iteration. From the second edition onward, the project included two iterations, as shown in Figure 2d. Figure 2e shows the timeline of the learning experience including the design thinking stages, two iterations, and learning activities. An elaborate explanation of the program, design stages, workshops, activities, and tools is provided in the Supporting Information. Tools for each stage are provided in a toolkit that is available: Design Thinking Toolkit.

To explain the learning experience, we can use the circular paint challenge. The first week the team met a contact person from the paint manufacturer for a briefing, and the students collected data by doing desk research. The students were asked to help the manufacturer produce circular paint by stimulating customers to return their leftovers. The students then took a few weeks to observe and interview paint users and conduct additional interviews with several other stakeholders. The team discovered that it is possible to make new high-quality paint from leftovers but to collect leftovers is a problem. Customers cannot just return them to the shop because by law domestic chemical waste must be delivered at a dedicated waste depot, and the threshold to do so is high. Customers tend to dump their cans in their ordinary containers when cleaning up their sheds. In reframing and ideation sessions, the team developed the idea of a “reverse vending machine”, so customers can return their paint to the shop where the employees do not have to touch the waste. The rest of the project the students sketched out their ideas, built models to show at hardware stores, and received feedback from stakeholders and customers to further develop and validate their idea.

### Constructive Alignment

**Figure 3:**
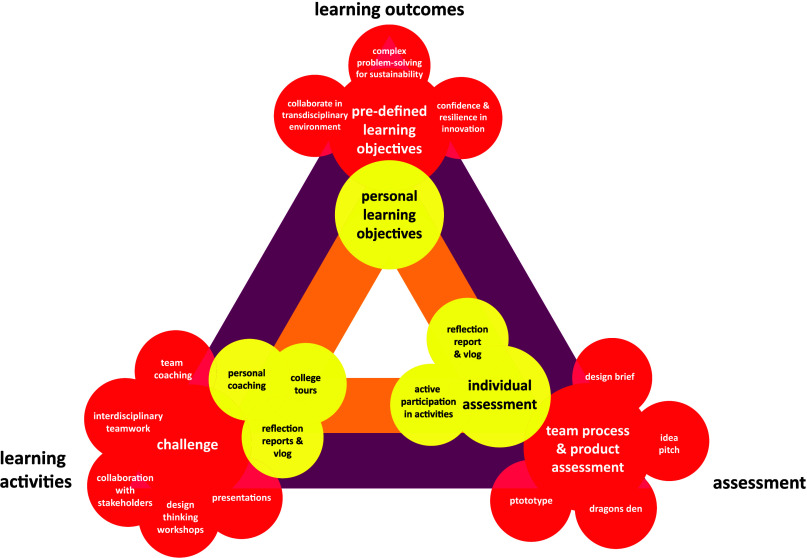
Constructive alignment within the Da Vinci Project. Two triangles can be distinguished. The purple/red triangle represents the predefined learning objectives and associated activities and team assessment. The orange/yellow triangle represents the alignment of personal learning objectives with activities as personal coaching and reflection, and with the individual assessment.

The application of the pedagogical principles of transdisciplinary education, CBL, and design thinking leads to a constructive alignment of learning outcomes, learning activities, and assessment. As shown in Figure 3, the alignment contains two levels. Predefined learning objectives are stimulated in activities tied to the challenge and assessed in team products, such as presentations of ideas and prototypes. The personal learning objectives are mainly stimulated by reflection reports and individual feedback from the mentor.

## Research

To discover if and how a combination of transdisciplinary CBL and design thinking facilitates development of sustainability change-makers competencies, we conducted educational design research, a systematic study of designing, developing, and evaluating educational interventions.^57^ The main question to be answered was whether and how the pedagogical principles of transdisciplinary education and CBL, combined with design thinking, provide an adequate framework for students to develop sustainability change-maker competencies. Before we could fulfill a benchmark, we answered the following questions first: What were the learning outcomes for the students participating in the Da Vinci Project between 2019 and 2023? What aspects of the program led to these learning outcomes?

### Data Collection and Analysis

**Figure 4:**
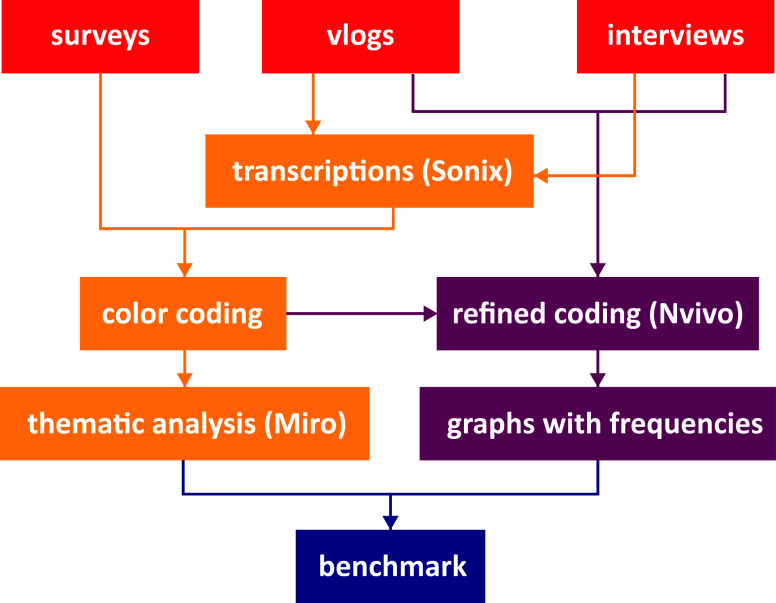
Flow of data analysis of surveys, vlogs, and interviews within the Da Vinci Project.

We started with an existing set of data: 46 reflection vlogs and 32 completed evaluation survey forms from the first four editions of the Da Vinci Project (Table 1). To complement these data, 19 semistructured interviews with students were conducted. All interviews and vlogs were transcribed with transcription software (Sonix). Color coding was used to label the relevant parts related to the research questions. All selected data from the interviews, vlogs, and surveys were collected and organized graphically on a Miro board. Then, a thematic analysis was done to analyze the diverse set of data. Themes were identified, and thematic maps were constructed to gain insights into the relationships between aspects of the program and self-perceived learning outcomes. Additionally, with an analysis of the interviews and vlogs in Nvivo, we further divided the self-perceived learning outcomes and the related program aspects into subcodes, and frequencies were analyzed. This enabled the authors to fulfill the benchmark with the competency frameworks of Figure 1. Furthermore, we completed the set of data with interviews with mentors (6) and contacts from partner organizations (4) to gain insight into their experience and add their perspective to our analysis. Their perspective is relevant to understand the student’s learning experience including their contribution and to gain insight into the value and drawbacks of the Da Vinci Project. The flow of analysis is shown in Figure 4. A more detailed description of the data collection and analysis is available in the Supporting Information.

**Table 1:** Data Collection (Vlogs and Interviews with Students) Divided by Edition

edition	pilot 2019–2020	2nd edition 2020–2021	3rd edition 2021–2022	4th edition 2022–2023	total
No. of students participating in project	21	31	25	14	91
No. of vlogs	*N* = 1	*N* = 10	*N* = 23	*N* = 12	*N* = 46
No. of respondents in survey	no survey	*N* = 17	*N* = 12	*N* = 3	*N* = 32
interviews with students	*N* = 15	*N* = 3	*N* = 1	*N* = 0	*N* = 19

## Findings

A variety of learning outcomes and related program aspects were mentioned in the interviews, vlogs and surveys. A logical explanation for this variety is that students pursued personal learning objectives. Consequently, we cannot conclude that all of the students developed the identified competencies. However, the thematic analysis yielded insights into which aspects were crucial in acquiring which learning outcomes. Moreover, we gained enough insight into the learning outcomes to fulfill a benchmark with the key competencies for sustainability and the IDGs. Furthermore, by analyzing frequencies, we uncovered which learning outcomes and which aspects play a significant role in the student’s learning experience in the Da Vinci Project. A more detailed presentation of the results, data analysis, including frequency analysis, benchmark, and thematic analysis is available in the Supporting Information.

### Thematic Analysis

**Figure 5:**
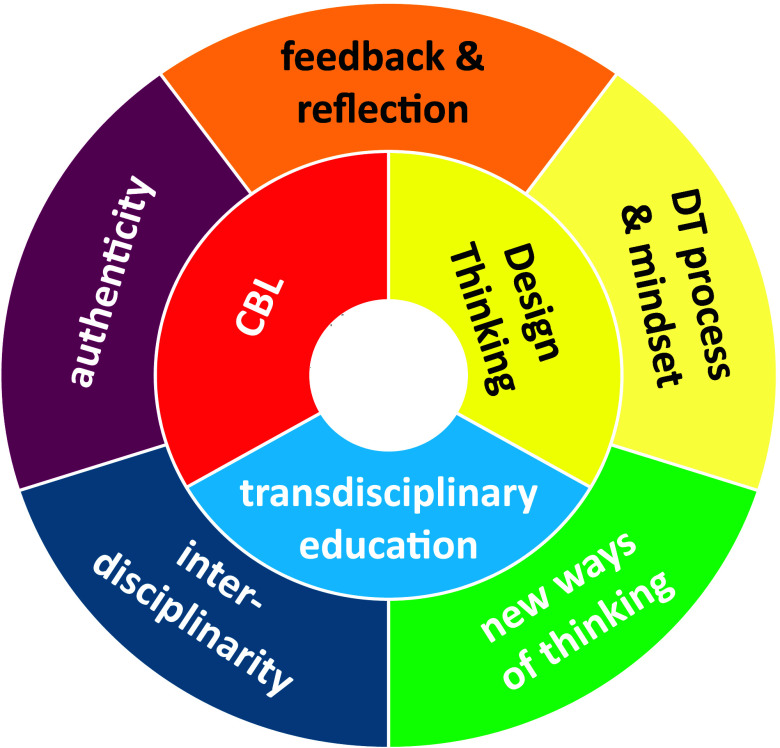
Themes identified in thematic analysis. The inner ring shows the pedagogies. The outer ring shows the themes by which the process of the student’s competency development can be explained.

Five main themes are identified that explain how students developed their learning outcomes in the Da Vinci Project. Here, we briefly discuss the themes, as also shown in Figure 5.

#### 1 Value of Interdisciplinarity

In the Da Vinci Project, students learned to value interdisciplinarity for sustainability problem-solving. Interdisciplinary teamwork stimulated students to gain insight in the value of their own discipline and to be more appreciative toward other scientific disciplines. The interdisciplinary environment in combination with design thinking exercises motivated an open attitude toward other people’s perspectives and opinions. Students learned how they can add value to sustainability problem-solving from their own discipline, and they become aware of the added value of an interdisciplinary team. They learned to tackle the challenge together, and some participants mentioned they also learned how to integrate different perspectives and disciplines to develop integrative solutions.

#### 2 New Ways of Thinking

The interdisciplinary environment in combination with design thinking stimulated new ways of thinking. Most students referred to creative thinking, critical thinking, and new approaches to problem-solving. Other new ways of thinking mentioned by participants included empathy, awareness of biases, and new learning strategies.

#### 3 Authentic Challenges

The authenticity of the challenge provoked major learnings. Students gained insight into the professional field of work they would not acquire in a simulated challenge. By working on real problems in collaboration with stakeholders, students became aware of the complexity of sustainability challenges. Furthermore, working with stakeholders on a real challenge incited the students’ motivation and engagement, which in turn enhances their learning in general.

#### 4 Design Thinking Process and Mindset, Creativity, And Creative Confidence

Design thinking workshops and applying the method, tools, and techniques on a real challenge stimulated (awareness of their own) creativity and (creative) confidence, which helped to navigate uncertainty and complexity. The iterative process reduced the fear of failure and stimulated resilience and perseverance. By applying design thinking, students developed the ability to structure the process of complex problem-solving with design thinking. Additionally, by improving their empathy skills, students could gain a deeper understanding of sustainability problems.

#### 5 Feedback and Reflection

Through frequent feedback on individual and team performance, students learned to cope with critical feedback and to understand that feedback is an opportunity to learn and improve. Feedback from the mentor on reflection reports and during team meetings encouraged students to improve their communication and collaboration skills. Some students gained the insight that conflict-avoiding behavior is detrimental to team performance and that addressing frustrations improves group dynamics. Reflection on personal development in reflection reports and during team meetings made students aware of their progression in achieving their personal learning goals. This stimulated self-awareness and personal leadership. Additionally, feedback from stakeholders and critical reflection on products enhanced the complexity-awareness and problem-solving capacities.

### Benchmark

The benchmark, shown in Table 2, indicates that a pedagogical framework of transdisciplinary CBL in combination with design thinking offers students opportunities to develop several sustainability change-maker competencies. Six out of eight key competencies for sustainable development were identified in the self-perceived learning outcomes. All five general academic competencies required for sustainability problem-solving have been determined, and we distinguished a majority of the IDGs.

**Table 2:** Benchmark of Self-Perceived Learning Outcomes with Sustainability Change-Maker Competencies

competency framework	clearly identified as learning outcome	identified as learning outcome in some cases	not identified
UNESCO/Wiek and Redman/Brundiers/Horn	interpersonal/collaboration competency	systems-thinking	futures-thinking/anticipatory competency
	intrapersonal competency/self-awareness	strategic-thinking/strategic competency	values-thinking/normative competency
	epistemic stability	implementation competency	
	epistemic adaptability	integrated problem-solving/integration competence	
general academic competencies	communication	learning	
	critical thinking	project management	
	creativity		
inner development goals (IDGs)	openness and learning mindset	complexity awareness	inner compass
	self-awareness	sense-making	integrity and authenticity
	critical thinking	trust	presence
	perspective skills	mobilization skills	long-term orientation and visioning
	empathy and compassion		appreciation
	communication skills		connectedness
	co-creation skills		humility
	inclusive mindset and intercultural competence		optimism
	courage		
	creativity		
	perseverance		

The fact that a majority of the IDGs was identified as learning outcomes is not surprising since the IDGs largely coincide with the characteristics of the design thinking mindset, which was provoked by the design thinking workshops and by applying the methodology on a challenge. There is also a clear connection between CBL and transdisciplinary education and the interpersonal, intrapersonal, and integration competence.

### Relation between Critical Aspects and Competency Development

**Figure 6:**
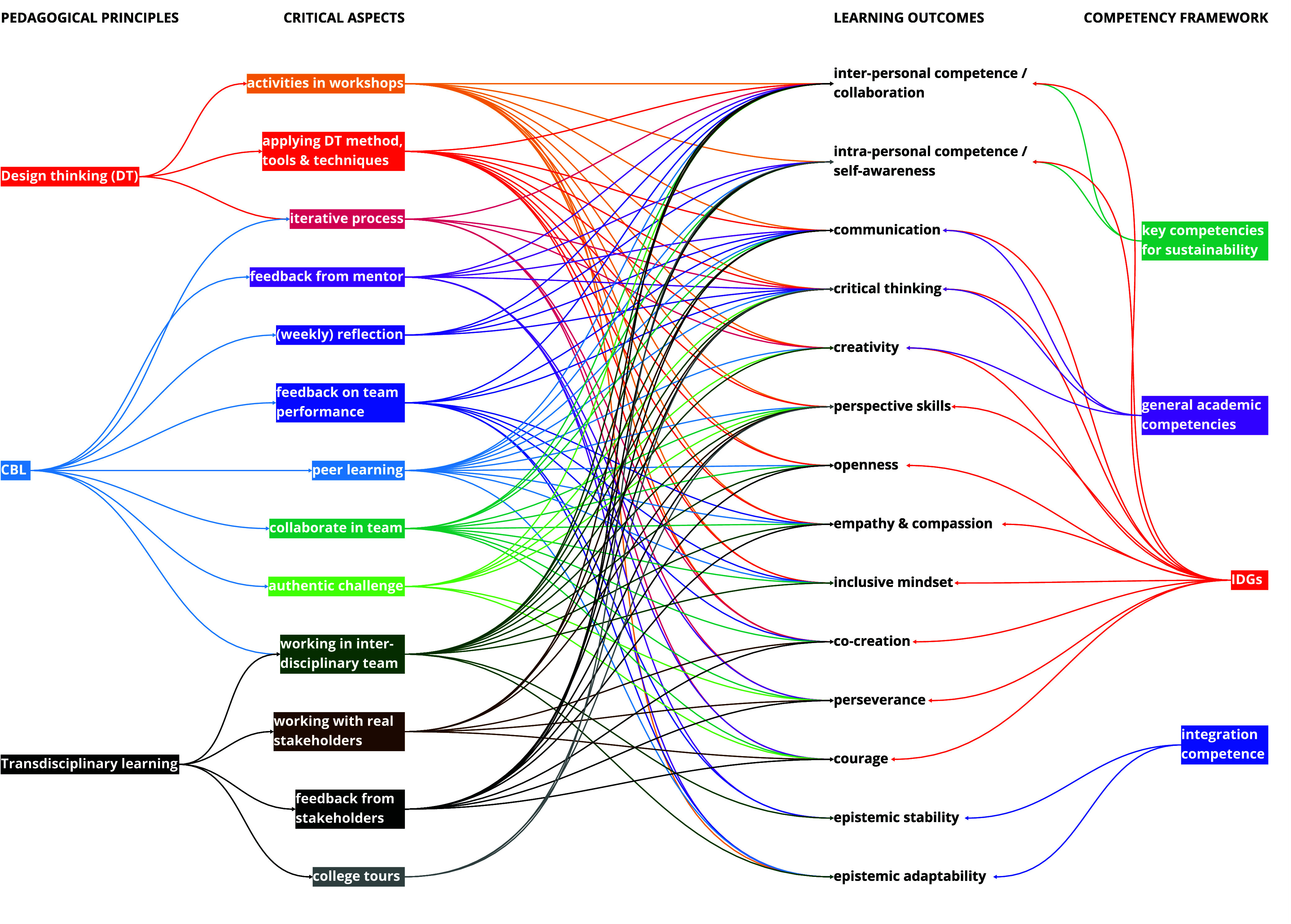
Relations between the critical aspects of the educational program and the learning outcomes that were clearly identified as sustainability change-maker competencies.

Further analysis provided insights in which aspects of the program where critical for the students’ development of sustainability change-maker competencies, as shown in Figure 6. This result was generated through a series of intermediate steps, further explained in the Supporting Information. The aspects playing a major role in competency development are workshop activities, applying design thinking, adapting an iterative process, coaching by a mentor, feedback, reflection, collaboration in an (interdisciplinary) team, peer learning, stakeholder involvement, a real challenge, and college tours. Figure 6 shows the complexity of the relations between critical aspects and learning outcomes. The students’ development of competencies depends on many critical aspects simultaneously. Other significant aspects that were also mentioned as decisive were the freedom to make their own decisions and safe space in the classroom and the team. These aspects are not included in Figure 6 because they are difficult to relate to one or more competencies but instead affect all learning.

## Discussion and Implications

Transdisciplinary CBL in combination with design thinking provides an adequate pedagogical framework to facilitate development of many sustainability change-makers competencies. We believe chemistry graduates will benefit from these competencies when applying the guiding principles of sustainable chemistry proposed by Blum et al.^7^ Graduates starting in the chemical industry can combine critical thinking and courage to address nonsustainable practices within the company, accompanied by a realistic view coming from complexity awareness. They can allocate their interpersonal skills to actively and strategically engage fellow employees, experts, divisions, and stakeholders outside to collaboratively make chemical products and even entire supply chains sustainable, but also desirable, viable, and feasible. They will benefit from empathy skills, openness, and perspective skills to understand how people perceive and use products like cosmetics, plastics, or paint and to cocreate systemic interventions for reuse, recycle, or upcycle materials. These future chemists can combine disciplinary with integrative skills, apply creativity, and guide iterative processes to develop benign chemicals and alternative solutions for problematic applications and to reduce impact of hazardous substances.

To those inspired to invest in a program such as the Da Vinci Project, we should also present the drawbacks learned through our inquiry. Table 3 presents the value and drawbacks of applying a combination of transdisciplinary challenge-based learning and design thinking to educate sustainability change-makers in an undergraduate extracurricular program, which we further explain in this paragraph.

**Table 3:** Value and Drawbacks of Applying a Combination of Transdisciplinary Challenge-Based Learning and Design Thinking to Foster Sustainability Change-Maker Competencies in an Undergraduate Extracurricular Program

value	drawbacks
1. Opportunity for students to develop many sustainability change-maker competencies, particularly interpersonal and intrapersonal competencies.	1. Full coverage of key competencies for sustainability is not possible in a short-term extracurricular program.
2. Constructive alignment between sustainability competencies and pedagogical principles of transdisciplinary challenge-based learning and design-thinking is adequate.	2. Assessment of transdisciplinary challenge-based learning is challenging because it is impossible to predeterminate learning outcomes.
3. Students can pursue their own learning objectives, followed by a process-based assessment.	3. Process-based assessment can be time-consuming and does not consider the quality of products, which is important for long-term engagement of stakeholders.
4. Students take ownership over learning process.	4. Students hesitate to reach out to stakeholders for feedback, which requires persistent mentors to encourage students.
5. Students learn to collaborate in interdisciplinary teams working on authentic challenges.	5. Requires teaching staff with advanced coaching skills and scaffolded learning activities for feedback literacy.
6. High level of student engagement due to real-life challenges and stakeholder involvement.	6. Organizing stakeholder involvement and real-life challenges are time-consuming.
7. Fosters integration competencies.	7. Requires teaching staff skilled in interdisciplinary learning to facilitate learning opportunities, i.e., addressing functional disagreement.
8. Design-thinking reinforces growth of change-maker competencies.	8. Requires teaching staff with design-thinking facilitation expertise.

This study shows that many valuable competencies can be developed in transdisciplinary CBL in combination with design thinking, but learning outcomes differ per student. Moreover, full coverage of sustainability change-maker competencies for all students cannot be expected in an extracurricular short-term program. Lack of coverage and integration of all competencies in a curriculum will inevitably lead to shortfalls in the undergraduate’s ability to tackle sustainability challenges.^27^

Alignment of competencies and pedagogical principles in the Da Vinci Project is adequate to develop change-maker competencies, but aligning assessment is challenging, especially with an interdisciplinary and multilevel group of students. The opportunity to pursue personal learning objectives, accompanied by frequent feedback and reflection, makes students active and engaged learners because they see their progression and they can pursue goals they personally value. However, evaluating personal learning objectives is difficult with traditional academic assessment methods. Process-based self-assessment or other reflective ways of assessment are more appropriate but can be time-consuming and do not consider the quality of the products students deliver. This quality is important, for example, for long-term engagement of partner organizations. Therefore, a combination of product-based and process-based assessment is advised^15^ as is done in the Da Vinci Project.

In line with theory on CBL,^18,19,23,33,35^ this study shows that students take ownership over their learning process, but it requires teachers with advanced coaching skills. When teachers are coaches instead of instructors, students learn to deal with freedom and responsibility to steer their own learning. Once they become aware that feedback is an opportunity to learn, they start to actively seek for feedback. However, students often remain hesitant to reach out to stakeholders despite encouragement by the mentor. Scaffolding feedback literacy based on the challenge-feedback-learning cycle^34^ can serve educators to develop learning activities.

As was expected from theory on transdisciplinary education,^6,30^ students developed skills such as collaboration, critical thinking, and empathy. The results also show that students develop epistemic stability and epistemic adaptability, which are both conditional for integrated problem-solving.^30^ One could argue whether second- and third-year bachelor students can already develop epistemic stability. However, students mentioned becoming more aware of their own discipline in interdisciplinary collaboration, appreciating their own discipline better, and learning what they could contribute to interdisciplinary problem-solving. We also detected functional disagreement, although it depended on the engagement of the mentor whether it resulted in actual learning. In line with theory on CBL, we saw that the students’ learning enhanced when mentors did not give answers but instead facilitated conversations about contrasting viewpoints, pointed out conflict-avoiding behavior, and encouraged students to address conflicts.

We can also conclude that design thinking had significant reinforcing effects on the development of change-maker competencies. With design thinking, disciplinary frames can be deconstructed, appreciation of other disciplines is fostered, cocreation skills are developed, and students learn to navigate complexity. Specifically, framing and creative thinking exercises motivate a pluralistic understanding of the problem and a tolerance toward the coexistence of multiple valid interpretations.^52−54^ Again, this requires skilled teaching staff to professionally facilitate a design thinking process. Additionally, an environment that stimulates a “designerly” mindset^39,46^ is indispensable for a profound learning experience.

Finally, we conclude that transdisciplinary CBL in combination with design thinking is beneficial for chemistry students. Chemistry teachers participating in the Da Vinci Project, for example, emphasized the important role of creativity in scientific breakthroughs in the interviews. While chemistry bachelor curricula usually do not stimulate students to explore radical ideas, students will likely hesitate to develop innovative experiments in the lab once they get to their thesis. Additionally, also drawn from the interviews, encouraging chemistry students to connect with other disciplines and the nonacademic world, provides them with a richer picture of the value of chemistry for society and career possibilities. Furthermore, engaging students in real-world problems involving nonacademic partners increases student engagement. However, developing, implementing, and organizing this type of education is more time-consuming and more expensive than traditional lectures and tests. The siloed structure of universities raises many hurdles to set up interdisciplinary programs. Moreover, engaging nonacademic partners maybe common in research, but not in education. There are usually no structural resources allocated to stakeholder engagement in education. Therefore, it took more than three years to set up a pilot, and after five years, the program unfortunately is still dependent on additional funding.

## Limitations

Though a large data set has been collected for this research and a broad analysis has been done, this study inevitably has limitations of which we here mention the most important ones.

- We did not study sustainability change-maker competency development in chemistry curricula. Additional research should be conducted to compare the learning outcomes of regular chemistry (under)graduates with those who additionally participated in change-maker education.
- This study cannot draw conclusions to whether the alumni of the Da Vinci Project will be successful sustainability change-makers in the future. Additional research should be done to understand whether the learning outcomes affect the graduates’ professional careers.
- We investigated the influence of program aspects on the learning outcomes, but we did not investigate the effects of individual teacher performance, while this was mentioned as critical by the mentors and some students. Therefore, different outcomes could be expected when teachers are being replaced or when this program design would be realized at another university.

## Conclusions and Outlook

Chemists can play a crucial role in sustainability transitions, but most chemistry graduates are not yet fully equipped to be change-makers. Performing in an interdisciplinary, multistakeholder context and navigating in the uncertainty and complexity of sustainability challenges requires advanced skills and attitudes that are not yet a self-evident part of chemistry curricula. In our research on the Da Vinci Project we learned that transdisciplinary CBL combined with design thinking is an adequate pedagogical framework to facilitate growth of sustainability change-maker competencies, such as empathy, creativity, collaborative teamwork, stakeholder engagement, and integrative problem-solving.^55−57^

On the other hand, teaching and organizing education, like the Da Vinci Project, is time-consuming, specific expertise and skills are required of teaching staff, and constructively aligning outcomes with assessment is difficult. Universities that encompass natural sciences, social sciences, and humanities are specifically suitable for this type of education because they enable an even broader diversity in interdisciplinary teams. Though there is no engineering faculty at Utrecht University and no engineering students who participated in our project, engineering students would be desirable participants, for they are likely to be equipped with prototyping skills.

The Da Vinci Project has been successful so far, but we continue to improve the learning experience and make it available to more students. One focus point is to improve the constructive alignment according to the latest scientific insights regarding sustainability change-maker competencies. Second, we focus on training (new) teaching staff to ensure the quality of coaching and workshops. Third, we will strengthen long-term relationships with societal partners to enable valuable learning experiences for both students and partners. What we have learned is that the results of the Da Vinci Project do not always meet the expectations of the societal partners. We assume that when collaboration is more intense and when students can spend more time on the challenge, the quality of the results will improve.

We used these learnings to extend our research to the Da Vinci Master Program, which started in September 2023. This program is based on the same pedagogical principles as the Da Vinci Project, but it is a 20-week full-time program, embedded in the curriculum of the Graduate School of Natural Sciences of Utrecht University. Here, extra attention is paid to cover and integrate key-competencies for sustainability, constructive alignment, and facilitation of integrated problem-solving. Moreover, this program is a collaboration with two other Dutch universities, one of which is a technical university. In the Da Vinci Master program, we will further investigate the students’ learning experience and the value for science and society. One of the challenges in the Master’s program is based on research on the *refinery of the future*, as recently published in *Nature*,^58^ in which students create a concept for a fossil-free refinery to make our goods from, e.g., municipal and agricultural waste, as well as carbon dioxide.

## Supporting Information Available

Elaboration of theoretical background including definitions of sustainability competencies • Elaborated explanation of program design. Description of workshops (including links to presentations), activities, and tools. Description of the editions of the Da Vinci Project from 2019 to 2024. Elaborated description of research process, data collection, and analysis. Elaborated description of findings. Elaborated version of limitations. Course manual for students. Course manual for mentors (PDF)
