# Supplementary material for: Da Vinci Project: Educating Sustainability Change-Makers with Transdisciplinary Challenge-Based Learning and Design Thinking
Source: J Chem Educ. 2024 Sep 17;101(10):4161–72. doi: 10.1021/acs.jchemed.4c00158 (PMC11465464; doi:10.1021/acs.jchemed.4c00158)
Supplement: Supplementary file 1 — ed4c00158_si_001.pdf [file ed4c00158_si_001.pdf]

Supporting information

The Da Vinci Project: Educating Sustainability Change-makers with Transdisciplinary Challenge-based Learning and Design Thinking

Fieke Sluijs,[1,*] Sabine G. Uijl,[2] Eelco T.C. Vogt,[1] and Bert M. Weckhuysen[1,*]

1. Inorganic Chemistry and Catalysis group, Institute for Sustainable and Circular Chemistry (ISCC), Department of Chemistry, Faculty of Science, Utrecht University, Universiteitsweg 99, 3584 CG Utrecht, The Netherlands
2. Alliance of TU/e, WUR, UU, and UMC Utrecht (EWUU), Princetonlaan 6, 3584 CB Utrecht, The Netherlands

Corresponding Authors
*E-mail: f.sluijs@uu.nl and b.m.weckhuysen@uu.nl

TABLE OF CONTENT

1 ELABORATION ON THEORETICAL BACKGROUND 3

2 ELABORATED EXPLANATION OF PROGRAM DESIGN..6

3 DESIGN THINKING WORKSHOPS, ACTIVITIES, AND TOOLS 9

4 FIVE EDITIONS OF THE DA VINCI PROJECT...13

5 RESEARCH PROCES & DATA COLLECTION 17

6 FINDINGS 20

7 THEMATIC ANALYSIS 26

8 ELABORATED VERSION OF LIMITATIONS 41

9 COURSE MANUAL (STUDENTS) 43

10 COURSE MANUAL (MENTORS)...55

REFERENCES...60

1 ELABORATION ON THEORETICAL BACKGROUND

Definitions of sustainability competencies

Table S1. Overview of key competencies for sustainability and definitions

Competency	Definition
Systems thinking competency	the abilities to recognize and understand relationships; to analyze complex systems; to think of how systems are embedded within different domains and different scales; and to deal with uncertainty.[i]
Futures-thinking competency (Brundiers, et al.; Wiek & Redman);<br>Anticipatory competency (UNESCO)	to be able to iterate and continuously refine one's own futures thinking (visions, scenarios, etc.), in productive and explicit tension to the status quo; recognizing the "implicitly held (and largely unrecognized) assumptions about how society works" and how they influence the status quo and critically reflecting how they might influence futures thinking.[ii]
Values-thinking Competency (Brundiers, et al.; Wiek & Redman);<br>Normative Competency (UNESCO)	the ability to differentiate between intrinsic and extrinsic values in the social and natural world to recognize normalized oppressive structures to identify and clarify one's own values; to explain how values are contextually, culturally, and historically reinforced; to critically evaluate how particular stated values align with agreed-upon sustainability values and to differentiate.[ii]
Strategic-thinking Competency (Brundiers, et al.; and Wiek & Redman)<br>Strategic competency (UNESCO)	The ability to recognize the historical roots and embedded resilience of deliberate and unintended unsustainability and the barriers to change; to creatively plan innovative experiments to test strategies.[ii]
Implementation Competency (Brundiers, et al., and Wiek & Redman);	the collective ability to realize a planned solution toward a sustainability-informed vision, to monitor and evaluate the realization process, and to address emerging challenges (adjustments), recognizing that sustainability problem-solving is a long-term, iterative process between planning, realization, and evaluation.[ii]
Integrated Problem-Solving Competency (Brundiers, et al., UNESCO);<br>Integration Competence (Wiek & Redman)	the ability to combine and integrate steps of the sustainability problem-solving process or competencies, while drawing on pertinent disciplinary, interdisciplinary, transdisciplinary, and other ways of knowing.[ii]
Inter-personal Competency (Brundiers, et al.; Wiek & Redman);<br>Collaboration Competency (UNESCO)	the ability to apply the concepts and methods of each competency not merely as "technical skills," but in ways that truly engage and motivate diverse stakeholders and to empathically work with collaborators' and citizens' different ways of knowing and communication.[ii]
Intra-personal Competency Brundiers, et al., and Wiek & Redman);<br>Self-Awareness (UNESCO)	the ability to be aware of one's own emotions, desires, thoughts, behaviors, and personality, as well as to regulate, motivate, and continually improve oneself drawing on competencies related to emotional intelligence and social and emotional learning.[ii]

Table S2. Overview of IDGs and definitions[iii]

Being:<br>Relationship to Self	Thinking:<br>Cognitive Skills	Relating:<br>Caring for Others and the World	Collaborating:<br>Social Skills	Acting:<br>Driving Change
Inner Compass:<br>Having a deeply felt sense of responsibility and commitment to values and purposes relating to the good of the whole	Critical thinking:<br>Skills in critically reviewing the validity of views, evidence, and plans	Appreciation:<br>Relating to others and to the world with a basic sense of appreciation, gratitude, and joy	Communication skills:<br>Ability to really listen to others, to foster genuine dialogue, to advocate own views skillfully, to manage conflicts constructively and to adapt communication to diverse groups	Courage:<br>Ability to stand up for values, make decisions, take decisive action and, if need be, challenge and disrupt existing structures and views
Integrity and Authenticity:<br>A commitment and ability to act with sincerity, honesty and integrity	Complexity awareness:<br>Understanding of and skills in working with complex and systemic conditions and casualties	Connectedness:<br>Having a keen sense of being connected with and/or being a part of a larger whole, such as a community, humanity or global ecosystem	Co-creation skills:<br>Skills and motivation to build, develop and facilitate collaborative relationships with diverse stake-holders, characterized by psychological safety and genuine co-creation	Creativity:<br>Ability to generate and develop original ideas, innovate and being willing to disrupt conventional patterns
Openness and Learning Mindset:<br>Having a basic mindset of curiosity and a willingness to be vulnerable and embrace change and grow	Perspective skills:<br>Skills in seeking, understanding and actively making use of insights from contrasting perspectives	Humility:<br>Being able to act in accordance with the needs of the situation, without concern for one's own importance	Inclusive mindset and Intercultural competence:<br>Willingness and competence to embrace diversity and include people and collectives with different views and backgrounds	Optimism:<br>Ability to sustain and communicate a sense of hope, positive attitude and confidence in the possibility of meaningful change
Self-awareness:<br>Ability to be in reflective contact with own thoughts, feelings and desires; having a realistic self-image and ability to regulate oneself	Sense-making:<br>Skills in seeing patterns, structuring the unknown and being able to consciously create stories	Empathy and Compassion:<br>Ability to relate to others, oneself and nature with kindness, empathy and compassion and the intention to address related suffering	Trust:<br>Ability to show trust and to create and maintain trusting relationships	Perseverance:<br>Ability to sustain engagement and remain determined and patient even when efforts take a long time to bear fruit
Presence:<br>Ability to be in the here and now, without judgement and in a state of open-ended presence	Long-term orientation and visioning:<br>Long-term orientation and ability to formulate and sustain commitment to visions relating to the larger context		Mobilization skills:<br>Skills in inspiring and mobilizing others to engage in shared purposes	

Challenged-based learning (CBL)

CBL has its roots in experiential learning, which can be defined broadly as the cognitive process of learning through experiences, narrowly defined as learning through reflection on doing.[iv] According to experiential learning theory, learning occurs in a cycle of subsequently concrete experience, reflective observation, abstract conceptualization, and active experimentation.[v] Meaningful learning occurs when students reflect on challenging experiences, triggered by the experiences themselves and by feedback.[vi] Effective learning, even for highly motivated students, depends on adequate feedback.[vii] The construction of meaning happens in a context of social interaction between students and teachers in which they share responsibility, so they both must develop feedback literacy. Students are responsible for engaging in the challenge and providing diagnostic information. Students must learn to actively seek for feedback information, respond, make sense, and use it.[viii]

Design Thinking

Multiple models have been developed that describe the design thinking methodology of which the Double Diamond of the Design Council (UK)[ix] and the five-step process of the Hasso Plattner Institute of Design,[x] also known as the Stanford d.School Model, are the most popular ones. Though different models use different terms for the stages, the process is similar. They all describe an alternation of divergent and convergent activities in continuous iteration to understand the problem and design a solution.

The highly reflective process of design does justice to the nature of CBL. In professional design practice, knowledge is gained through 'reflection-in-action'.[xi,xii] This way, new knowledge is created by iteratively designing artifacts. The representations of design ideas are examples of 'epistemic artifacts', cognitive objects created to explain theories, validate hypotheses, and solve problems.[xiii]

2 ELABORATED EXPLANATION OF PROGRAM DESIGN

The Da Vinci Project immerses students in interdisciplinary teams, tackling sustainability challenges alongside societal partners. While teams enjoy much freedom to explore and solve problems, regular interaction with partners is crucial for information gathering, alignment, and feedback. This collaboration extends to engaging other stakeholders, conducting interviews with end-users, and seeking advice from experts. For most undergraduates, it is the first time to collaborate with stakeholders beyond the university. Teams are supported by mentors from academic staff who assume coaching roles.

Reflecting on and receiving feedback are integral components of the project. This occurs across two dimensions: personal development and team performance. Students articulate personal learning objectives in the first week and then submit reflections on their learning experience and personal performance weekly. The mentor provides individual feedback on these reflections. For students, the reflections and feedback serve as tools for self-awareness and ownership of their learning process, while mentors utilize them to guide individual and team performance. At the project's conclusion, students submit a vlog in which they reflect on the entire learning experience.

Active seeking of feedback from external stakeholders is encouraged to enhance results, complemented by three formal feedback events. The first is a *dragon's den* with external professionals providing critical feedback on solution pitches. To prepare students for receiving critical feedback they learn to pitch in less formal meetings. Additionally, embodied learning is used to reinforce resilience to critical feedback. In a Taekwondo workshop, parallels are drawn with the physical reaction to an attack. In the second formal event, the *expert meeting*, sustainability experts collaborate with teams to enhance prototypes' sustainability, feasibility, viability, and desirability. Lastly, the *prototype party* marks the program's conclusion, celebrating achievements and showcasing final prototypes to stakeholders and peers.

In design thinking workshops, students learn to apply design thinking to their challenge. The students start *Empathizing* with stakeholders and end-users by conducting qualitative research, gaining a deeper understanding of the problems beyond the surface.

They practice empathy skills in activities during the workshops, which improves collaboration in these diverse teams. Empathy skills are crucial to establish psychological safety, to deal with uncertainty and complexity involved in sustainability challenges, and to drive innovation.[ii] Although it is defined as a separate step, empathy will remain important throughout the whole process. In the *Define* stage, students make sense of the diverse dataset, identifying insights and articulating problem statements. The *Frame* stage involves exploring multiple perspectives on the problem, acknowledging their complex nature. *Ideate* follows, where students generate ideas and develop creative concepts. One concept is selected for further development into a tangible *Prototype* in collaboration with the societal partner. The *Test* stage involves prototype testing and feedback reception. The dragon's den marks the end of the first iteration, planned halfway in the program. Then, a second iteration follows. Tools for each stage are provided in the workshops and a toolkit available on: Design Thinking Toolkit. An overview of stages, activities, and tools is provided in the Supporting Information.

Learning objectives & assessment

At the end of the program students have the:

- Ability to collaborate in a transdisciplinary environment and in a multiple stakeholder context.
- Ability to navigate in the uncertainty and complexity of the problem-solution space of sustainability challenges.
- Creative confidence and the resilience to drive innovation.

These general learning outcomes are further articulated in twelve learning objectives.
At the end of the program, students can:

1. Conduct a sustainability problem analysis from a human-centered perspective.
2. Frame insights from qualitative research in multiple inspiring ways.
3. Adapt a divergent thinking mode to generate a vast number of ideas for solutions.
4. Develop several distinctive creative concepts for solutions to solve the problems regarding the sustainability challenge.
5. Develop tangible prototypes to test assumptions about the proposed solutions.
6. Adapt the prototype according to the feedback from testing.

7. Reflect on personal skills, insights, and behavior in relation to the problem-solving process in an interdisciplinary team.
8. Provide and receive feedback.
9. Contribute to client's goals.
10. Take initiative to steer personal development and to enhance team performance.
11. Think critically of the process of sustainability problem-solving in an interdisciplinary team.

Assessment procedure

Students are assessed on pre-defined objectives and personal learning objectives. A set of pre-defined learning objectives on product and process level are articulated and operationalized in a rubric for assessment. To pass the assessment, students show their growth in competence in required deliverables. The deliverables of the teamwork consist of a design brief, a frame board presentation, a prototype presentation at the dragon's den and the final presentation at the prototype party. The deliverables of the individual assessment are the weekly reflection reports and the final reflection vlog. The mentor is the examinator of both the team and the individual work. The assessment rubrics can be found in the course manual for students (chapter 9).

3 DESIGN THINKING WORKSHOPS, ACTIVITIES, AND TOOLS

Design Thinking Workshops

During the design thinking workshops students engage in design thinking activities to develop skills and attitudes beneficial for resolving sustainability problems. The workshops are aligned with the stages of the design thinking process: empathize, define, frame, ideate, prototype, and test.

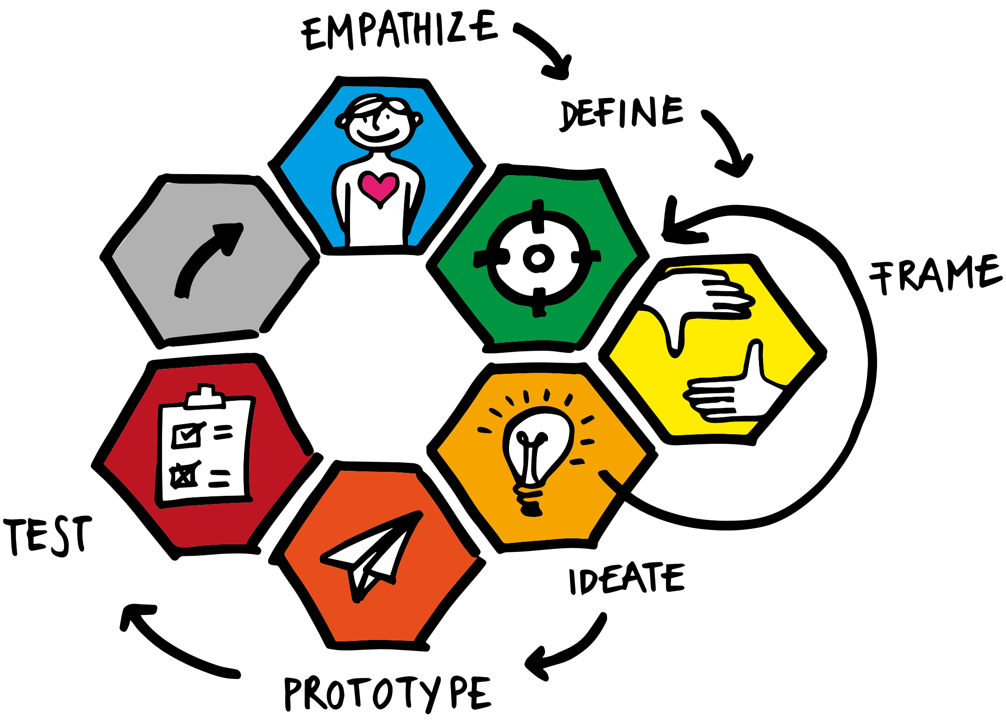

Visual of design thinking process included in slides of the design thinking workshops

The presentations of the workshops, which include instructions for activities, are available on SlideShare:

Workshop 1: Introduction

Workshop 2: Discover

Workshop 3: Empathy

Workshop 4: Define & Reframe

Workshop 5: Ideate

Workshop 6: Visual Storytelling

Workshop 7: Prototyping

Workshop 8: Creative Confidence

Stages, activities, tools, and techniques

Table S3 provides an overview of activities, and tools applied. A brief description of the activities students engage in during their challenge is presented below in Table 4. A complete overview of the tools is available in the online toolkit.

Table S3. Overview design thinking stages, activities, and tools in the Da Vinci Project

Design Thinking Stages	Activities	Tools & Techniques
Start project	Challenge Brief & Debrief	Debrief format
Empathize	Immersion in challenge<br>Empathize with end-user<br>Field research<br>Desk research	Ethnographic research<br>Observation<br>1-on-1 Interview
Define	Identifying insights<br>Sense-making<br>Triangulation<br>Problem analysis	Stakeholder Map<br>Context Map<br>Journey Map<br>Persona<br>Problem Statements
Frame	Framing and Reframing<br>Pitching Frame Boards	Framing techniques, e.g. How might we-questions, POV, Alter Ego's<br>Frame Board
Ideate	Organizing brainstorm sessions<br>Generating ideas<br>Brainstorm<br>Sketching and other visualization<br>Concept creation	Lightning Talks<br>Ideate techniques, e.g. Idea Purge, Reverse Brainstorm, Lotus Blossom,<br>Visualization techniques, e.g. Crazy 8's, Storyboard
Prototype	Develop Prototype Strategy<br>Rapid Prototyping<br>Create Prototypes	Prototype Strategy Map<br>Prototyping techniques, e.g. Paper prototype, Mock-ups, 3D modelling
Test	Setting up test/experiment<br>Recruit test participants<br>Testing prototype<br>Capture feedback	Testing techniques, e.g. Role Play, Wizard of Oz, A/B Test, Usability test, Pitch<br>Feedback Capture Grid

Table S4. Description of activities per stage

Stage	Description of activities
Start project	Challenge Brief & Debrief:<br>Students meet the contact person(s) from the societal partner for a briefing on the challenge and they read up on information regarding the project. Then they create a debrief to define the scope and get feedback on it from the contact person.
Empathize	Students do desk-research, conduct interviews with experts and stakeholders to collect insights on the problems involving the challenge. They conduct ethnographic research (observations, interviews) to gain insights in the characteristics, needs and behavior of the people they will design solutions for.
Define	Students identify insights in sense-making sessions by mapping the research data in stakeholder maps, context maps, and affinity maps.<br>They identify patterns in user characteristics, needs and behavior with journey mapping and personas. In the sense-making sessions students identify multiple problems involving the challenge and articulate problem statements.
Frame	Students explore multiple perspectives on the problems involving challenge with framing and reframing techniques. They create frame boards to articulate and explore multiple perspectives and they choose the most promising frames as a starting point for ideation.
Ideation	Students organize creative sessions to generate a vast number of ideas to solve the problems detected in the challenge. They use brainstorming and other creative techniques such as visualization for ideation and to create coherent innovative concepts. They gather inspiration and inspire each other with lightning talks. A frame board pitch marks the final step of ideation, when students present multiple concepts, visually elaborated frame boards. With the feedback from mentors, peers and their commissioning partner, the students adapt the concepts and select the best ideas to follow up on.
Prototyping	Students start the prototyping stage with sketching their ideas and creating storyboards to figure out the interaction of the end-user with the solution. They use rapid prototyping techniques to make the ideas tangible, to align and to learn what works and what doesn't. Students think through a strategy to test the concept with the end-user. They figure out what is the most critical assumption and how to validate whether that assumption is true or false with an experiment.
Test	Students set up experiments to learn whether their ideas for solutions are in the right direction. They need to recruit test participants. They create the prototype the participants can interact with, they conduct the experiment, and capture feedback. The feedback can be used to adapt the prototype and/or to do additional research, reframing, and ideation.

Constructive alignment

Table S5 Constructive alignment of the Da Vinci Project

Learning outcomes	Learning activities	Assessment
Pre-defined learning objectives:<br>- Ability to collaborate in a transdisciplinary environment and in a multiple stakeholder context.<br>- Ability to navigate in the uncertainty and complexity of the problem-solution space of sustainability challenges.<br>- Creative confidence and the resilience to drive innovation.	- Challenge<br>- Interdisciplinary teamwork<br>- Collaboration with stakeholders<br>- Team coaching<br>- Design thinking workshops<br>- College tours<br>- Presentations & feedback<br>- Expert meeting<br>- Taekwondo	Team Assessment:<br>- Design Brief<br>- Idea pitch<br>- Dragon's den<br>- Prototype
Personal learning objectives	- Writing reflections reports<br>- Creating reflection vlog<br>- College tours<br>- Personal coaching	Individual assessment:<br>- Active participating in activities<br>- Reflections reports (weekly)<br>- Reflection vlog

4 FIVE EDITIONS OF THE DA VINCI PROJECT

From 2019 to 2024, five editions of the Da Vinci Project took place yearly and 113 students from more than 30 different Bachelor courses participated. Figure S1 and Table S6 show the share per bachelor's degree.

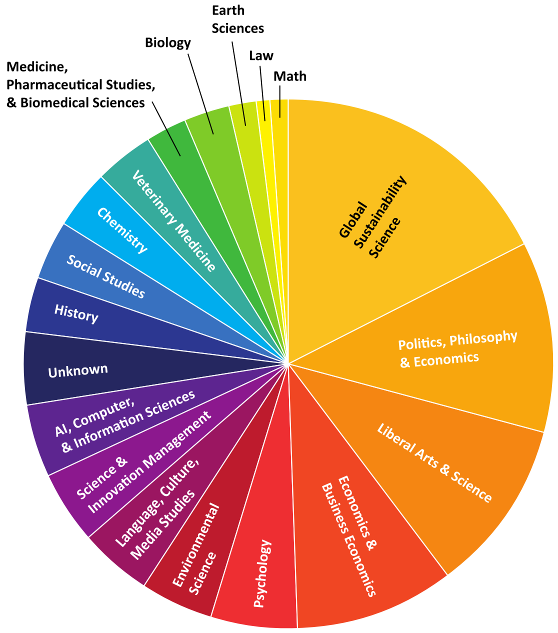

Figure S1. Diagram showing the share of students per bachelor's degree. Science students are underrepresented, whereas GSS, PPE, LAS and Economics students are overrepresented.

Table S6. Share of Bachelor's degree per edition

Bachelor's degree	Pilot	2nd	3rd	4th	5th	Total
GSS	2	1	1	5	11	20
PPE	2	4	3	2	2	13
LAS	2	6	2	1	1	12
Economics & Business Economics	2	1	3		4	11
Psychology	2	3	1			6
Environmental Sciences			4	1		5
Language /Culture/Media Studies	3	1	1			5
Science & Innovation Management	1	1	2	1		5
AI/Computer/Information Science	2	1	2			5
History	2	1			1	4
Social Studies	1	1		1	1	4
Chemistry	1		1	1	1	4
Veterinary Medicine	1	3				4
Medicine/Pharmaceutical/Biomedical Science			2	1		3
Biology		1	2			3
Earth Sciences		1		1		2
Law		1				1
Math		1				1
unknown		3	1		1	5
Total	21	31	25	14	22	113

The pilot (2019-2020)

The pilot of the Da Vinci Project took 12 weeks and 21 students participated. The program involved 32 contact hours (including 15h of design thinking workshops, 6h of presentation and feedback sessions, a 5 h fieldtrip, a 3 h dragon's den and a 3 h prototype party) and 178 designated self-study hours.

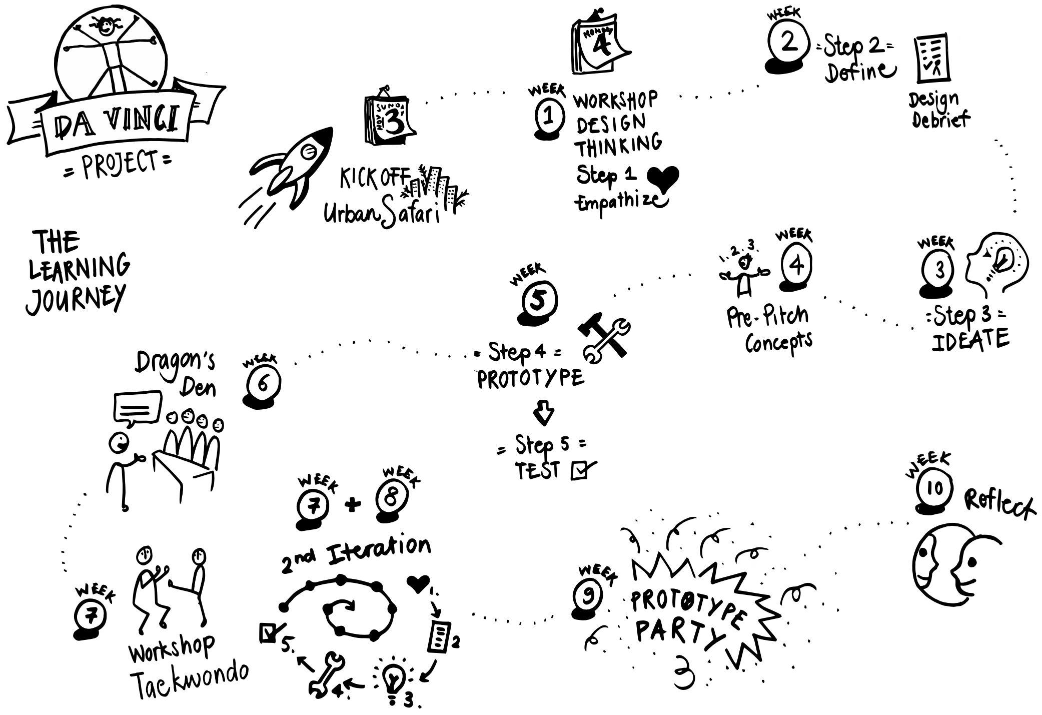

Visual of learning journey Da Vinci Project pilot edition.

Active Learning Classroom

Most of the meetings in the pilot edition, as well as in the fourth and fifth edition, took place in a room with special features, the Active Learning Classroom (ALC), one of the *future learning spaces* available at the Utrecht University. This classroom is designed based on the principles of the ALC to create a learning environment in support of active learning pedagogy and collective problem solving.[iii] The ALC differs from a lecture hall and stimulates active participation of students in several ways. There is no clear division between front and back, which breaks down the traditional hierarchy and increases the mobility for the instructor. Seating around tables allow students to face each other and thus support small-group work. Tables are paired with their own whiteboards for visualization. Tables are linked to large LCD displays so students can project their computer screens to the group and a teacher can choose a table's work to share with the entire class.[v]

Modifications after pilot

The program was adapted after the evaluation of the pilot. The **second** (2020-2021), **third** (2021-2022), **fourth** (2022-2023), and **fifth edition** (2023-2024) were extended to twenty weeks, 84 contact hours (including 40 h of group work and meetings with mentor, 24 h of design thinking workshops, 6 h of presentation and feedback sessions, a 5 h fieldtrip, a 3 h dragon's den, a 3 h prototype party and 6 h college tours) and 196 h designated self-study hours. The first main difference to the pilot is the extra time to develop and test the prototypes. The second main difference is the additional designated hours for group work and meetings with the mentor. During the pilot, the mentor was present at the design thinking workshops. Other meetings were initiated by the students themselves and the mentor didn't attend these. The last main difference was the enhancement of the program with *college tours,* meetings with influential change-makers in the field of sustainability development. The idea of the college tours is borrowed from a Dutch television show in which influential professionals are being interviewed by students.

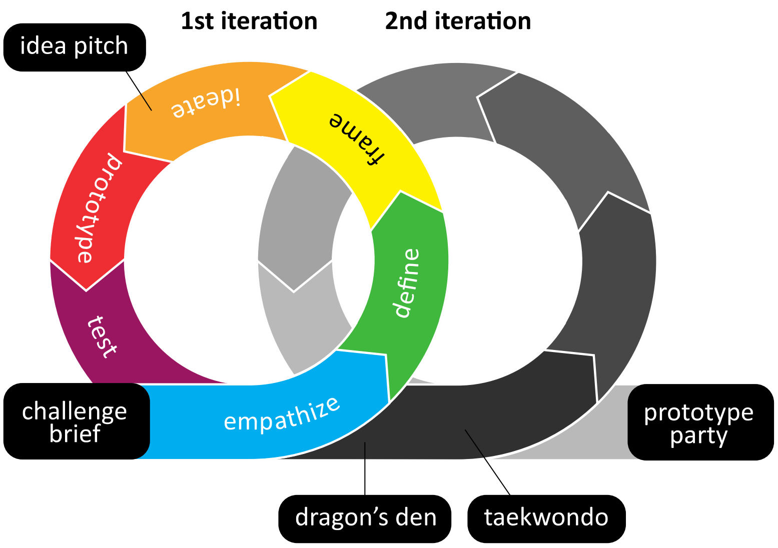

Visual of learning journey fifth edition.

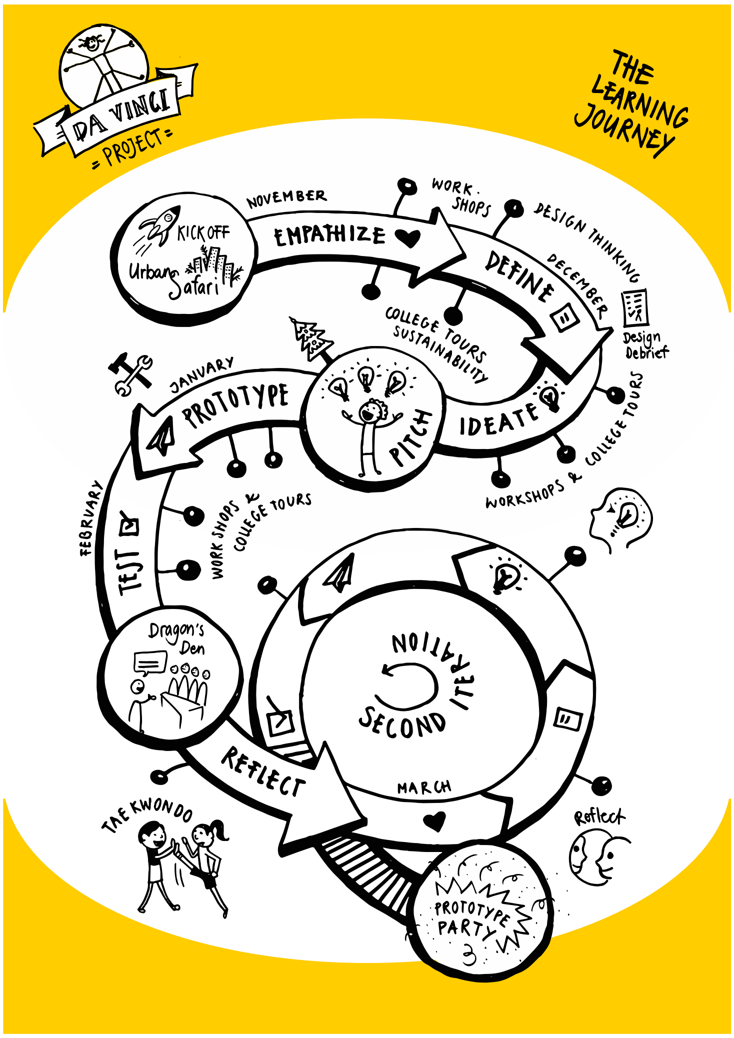

Visual of learning journey second and third edition.

COVID-19 editions of the Da Vinci Project

The **second** and **third edition** of the program needed to be adapted according to COVID-19 regulations. All meetings during the pilot took place in person. Halfway the second edition we switched to online learning environments. MS Teams, Zoom and Mural were used to facilitate the workshops, presentations, and events. A part of the meetings in the third edition also took place completely online. Most workshops were facilitated as hybrid classrooms with a focus on being activities on location, but with online facilities for participants in quarantine. We are aware that the way of facilitation (online, offline or hybrid) influences the learning outcomes. The purpose of this study is not to compare the editions, nor to discover differences in learning outcomes between offline, online, and hybrid activities, but to explore the learning outcomes for all editions.

5 RESEARCH PROCES & DATA COLLECTION

In 2018 and 2019 the Da Vinci Project was designed, which resulted in the pilot taking place in the second half of 2019. After every edition, evaluation took place and further development of the program followed. For a more in-depth inquiry of learning outcomes and related program aspects, analysis of surveys and vlogs was done, and additionally interviews were conducted and analyzed. In Figure S2, a timeline of the research process is presented.

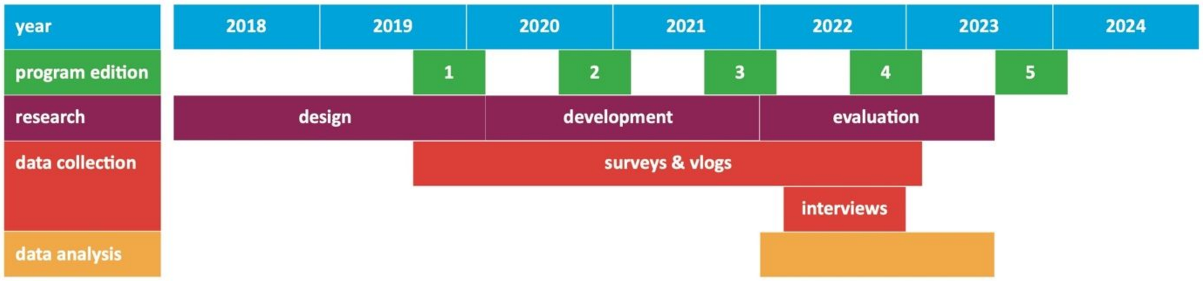

Figure S2. Timeline of Da Vinci Project, program editions and research activities.

We started the analysis with an existing set of data: 46 reflection vlogs and 32 completed evaluation survey forms. In the vlogs students reflect on their learning experience in the Da Vinci Project. There were no detailed instructions how to do the vlog, except for the following criteria for assessment:

- The reflection explains the student's thinking about his/her own learning processes.
- The reflection critically analyses the learning experience.
- The reflection articulates connections between this learning experience and content from other courses, past learning experiences and/or future goals.

No survey was conducted in the pilot. The second and third edition was evaluated by a survey with open questions about what aspects they liked and what could be improved about the Da Vinci Project generally and the workshops, college tours, dragon's den, and prototype party. The questionnaire finished with an open field for all other comments. In the survey of the 4th edition a new question was added: *What is the most important thing you learned in the Da Vinci Project? Please, tell us why.*

To complement the data, we conducted 19 interviews with students. Because there were hardly any data from the pilot, most of the interviews were done with students from

the pilot, see Table S7. A selection of five students from the second edition were invited for an interview, of which three responded. The selection was based on the criterium of disciplinary diversity. In the third edition only a chemistry student was interviewed.

Table S7. Data collection

Surveys	Vlogs	Interviews with students	Interviews with staff	Interviews with partners
32	46	19	6	4

The interviews are semi-structured, the main questions for the students were:

- How did you experience the Da Vinci Project?
- Why did you register?
- What did you learn?
- What is the most important learning outcome for you?
- How valuable is that learning outcome to you?
- What aspects in the Da Vinci Project led to these learning outcomes?

Data Analysis

The interviews and vlogs were transcribed with transcription software (Sonix). Color coding was used to identify and label relevant parts related to the research questions. Color codes were:

1. Personal experience of the program (pink)
2. Key learnings (red)
3. Why are these learning important for the student? (orange)
4. How did the student learn this? Aspects of the program (yellow)
5. Personal value and longer lasting impact of program (green)
6. Differences to other educational programs (blue)
7. Other interesting remarks (purple)

Color coding was used to identify and label relevant parts related to the research questions. All selected data from the interviews, vlogs, and surveys were collected and graphically organized on a Miro board. Then, a thematic analysis was done to analyze the divers set of data. Themes were identified, and a thematic map was constructed to gain insights in the relationships between aspects of the program and learning outcomes. The thematic map are available through this link: Thematic Analysis Da Vinci Project.

Additionally, with an analysis of the interviews (19) and vlogs (44) in Nvivo we further refined the self-perceived learning outcomes and the related program aspects into subcodes and frequencies were analyzed.

6 FINDINGS

Self-perceived learning outcomes

Students mentioned a broad set of learnings which can be divided in three categories: knowledge & insights, skills, and mindset & attitude. The table below presents all the self-perceived learnings mentioned by students in the interviews, vlogs, and surveys, after eliminating similar terms, such as 'creative thinking' and 'out-of-the-box thinking'.

Table S8. Categorized list of learning outcomes mentioned by students in vlogs, interviews and surveys

Knowledge & insights	Skills	Mindset & attitude
• Problem-solving process<br>• Research<br>• Design Thinking<br>• Human-centered design<br>• Creativity<br>• Team dynamics<br>• Value of interdisciplinarity<br>• Sustainability aspects specific to challenge<br>• Complexity of sustainability<br>• Behavioral aspects in sustainability<br>• Business/Organizational processes<br>• Insights in the industry<br>• Insights in the professional field of work<br>• Future work opportunities	• Problem-solving skills<br>• Design (thinking) skills<br>• Empathy skills<br>• Framing<br>• Apply creative techniques<br>• Brainstorming<br>• Visualization<br>• Creating a prototype<br>• Creative thinking<br>• Critical thinking<br>• Collaborating with stakeholders<br>• Collaborating in team<br>• Work in interdisciplinary team<br>• Reflection<br>• Personal leadership skill<br>• Giving and receiving feedback<br>• Communication<br>• Presenting<br>• Conducting interviews<br>• Leadership skills<br>• Learning skills	• Empathy<br>• Creativity<br>• Confidence<br>• Dealing with uncertainty<br>• Comfortable with ambiguity<br>• Resilience<br>• Perseverance<br>• Open towards others<br>• Trust<br>• Process focused instead of output focused<br>• Learn from failure<br>• Awareness of biases<br>• Patience<br>• Thinking in possibilities instead of limitations<br>•<br>•

Frequencies of self-perceived learning outcomes: interviews and vlogs (N=63)

The data collection analyzed in Nvivo consisted of 44 vlogs and 19 interviews. Originally, we had 46 vlogs, but we eliminated two vlogs because we also had an interview with these students. We analyzed the number of students mentioning learning outcomes in the three

categories: knowledge & insights, skills, and mindset & attitude. We also analyzed how many students mentioned a certain type within these categories.

In the vlogs and in the interviews, 52 out of 63 students mentioned one or more specific skills as important learning outcome. Interdisciplinary collaboration (24), collaboration in a team (19), creative thinking (27), and other design skills (31) are most frequently mentioned, as shown in Figure S3a. 31 students mentioned learning outcomes in the category of mindset & attitude. As shown in Figure S3b, empathy and openness (10), dealing with uncertainty and ambiguity (8), and other characteristics of the design thinking mindset (11) are most frequently mentioned. 28 students mentioned learning outcomes in the category of knowledge & insights. Knowledge and insights about design thinking (11) and sustainability (11) are most frequently mentioned (Figure S3c).

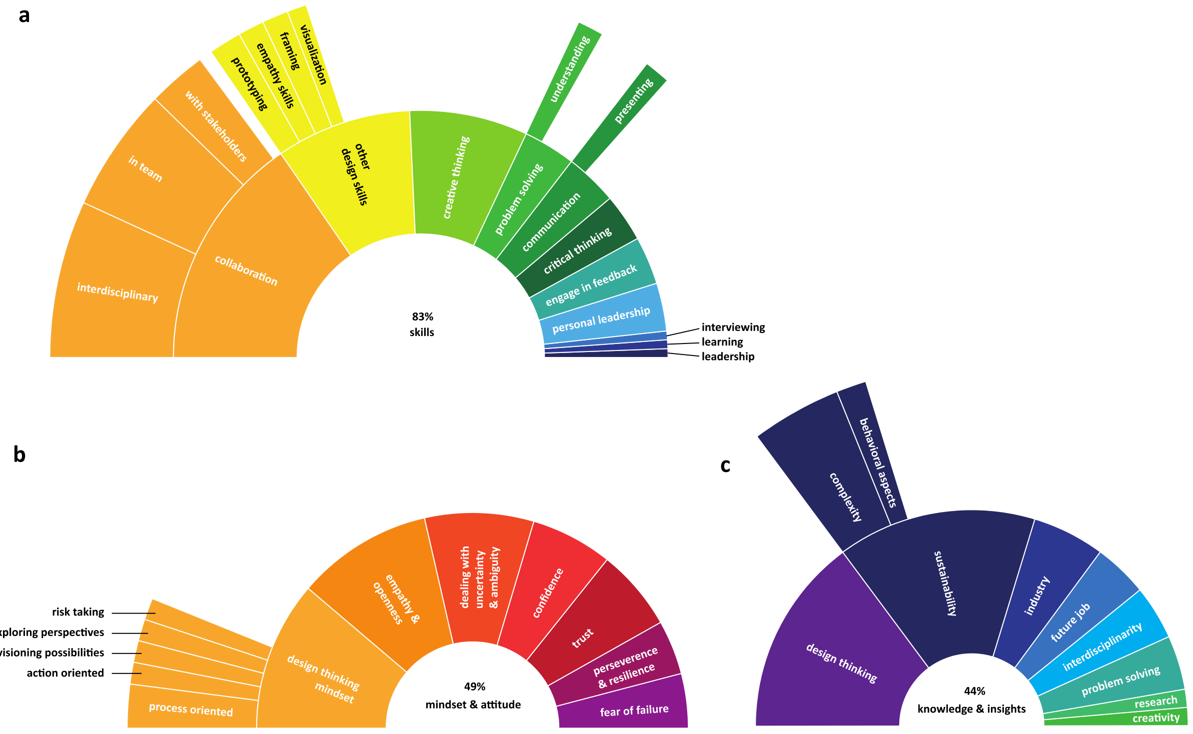

Figure S3. Share of specific learning outcomes mentioned in vlogs and interviews, divided in three categories: skills (a), mindset & attitude (b), and knowledge & insights (c). 83% of the students mentioned one or more learning outcomes in the skills category, 49% mentioned one or more learning in the mindset & attitude category, and 44% of the students mentioned learning outcomes in the category of knowledge & insights. Collaboration (62%), creative thinking (43%), and other design skills (49%) are most frequently mentioned skills (Figure S3a). Empathy and openness (16%), dealing with uncertainty and ambiguity (13%), and other abilities associated with the design thinking mindset (17%) are most frequently mentioned in the mindset/attitude category (Figure S3b). Design thinking (17%) and sustainability (16%) are the most frequently mentioned domains of knowledge and insights (Figure S3c).

Analysis of frequencies learning outcomes in further detail

In this section we show more detailed numbers from our analysis of learning outcomes in Nvivo.

Table S9. Number of students mentioning learning outcomes per category

Category	Absolute: number of students mentioned outcomes in this category	Percentage of students mentioning this category
Skills	52	83%
Attitude	31	49%
Knowledge	28	53%
		100%

Table S10. Number of students per specific learning outcome in the category of knowledge & insights

Knowledge & Insights	Subcodes	Number of students mentioning this type of knowledge and/or insight
Creativity		1
Research		1
Problem solving		3
Future work opportunities		3
Value of interdisciplinarity		3
Organizations & industry		4
Sustainability		10
	Behavioral aspects	1
	Complexity	3
Design thinking		11

Table S11. Number of students per specific learning outcome in the category of mindset & attitude

Mindset & attitude	Subcodes	Number of students mentioning this mindset and/or attitude
Perseverance/Resilience		4
Trust		6
Confidence		6
Reduction fear of failure		4
Empathy/Openness		10
Dealing with uncertainty/ambiguity		8
Other/general DT mindset		11
	Process oriented	2
	Action oriented	1
	Envisioning possibilities	1
	Exploring perspectives	1
	Risk taking	1

Table S12. Number of students per specific learning outcome in the category of skills

Skills	Subcodes	Number of students mentioning this skill
Research		2
	Interviewing	2

Collaboration		39
	with stakeholders	9
	in team	19
	interdisciplinary	24
Learning		2
Leadership		2
Communication		12
	Presentation	4
Personal leadership		11
Problem solving		10
	Problem understanding	4
Critical thinking		11
Engage in feedback		11
Creative thinking		27
Other design skills		21
	Visualization	3
	Framing	4
	Empathy skills	4
	Prototyping	5

Aspects responsible for learning outcomes

In the interviews and vlogs, students were asked to tell which aspects were responsible for their learning outcomes. Students mentioned a wide range of aspects that stimulated their learnings. It varies from specific activities during workshops to atmospheric features and characteristics of interaction between people. 55 out of 63 students mentioned CBL-related aspects, such as stakeholder involvement and working on a real-life challenge, as shown in Figure S4. Other decisive stimulators were social aspects (the student team and the whole group), the design thinking workshops, and the interdisciplinary learning environment. The exact numbers are shown in Table S13.

As shown in Table S14, in our analysis of the interviews, vlogs, and surveys we also distinguished the aspects we planned by design from the aspects that emerged from it. The planned aspects can further be divided into four categories: aspects related to the challenge, aspects related to the team and mentoring, general program aspects and aspects directly related to design thinking. The emerging aspects cannot be assigned to specific aspects since multiple planned aspects are always responsible for one emerging aspect and emerging aspects can stimulate other emerging aspects.

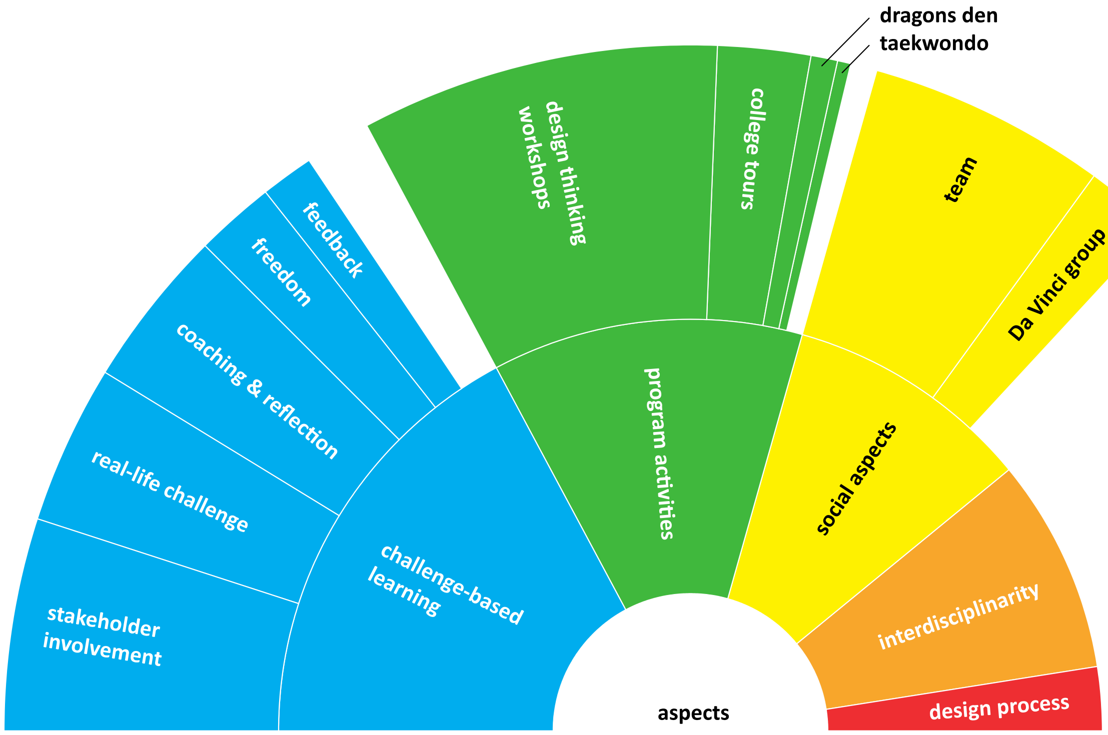

Figure S4. Share of specific aspects mentioned by students regarding their learning outcomes. Aspects associated with challenge-based learning are most frequently mentioned (87%). Other decisive stimulators are social environment (49%), the design thinking workshops (43%), and the interdisciplinary environment (43%).

Table S13. Number of students mentioning specific aspects as decisive for their learning outcomes in interviews and vlogs

Aspects	Subcodes	Number of students mentioning aspects in this category or subcategory
CBL		55
	Stakeholder involvement	16
	Feedback	4
	Freedom	6
	Real-life challenge	12
	Coaching & reflection	12
Design Process		8
Interdisciplinarity		27
Program activities		39
	College tours	7
	Dragon's den	2
	DT workshops	27
	Taekwondo	1
Social aspects		31
	Team	18
	Da Vinci group	6

Table S14. Aspects mentioned by students as decisive for their learning outcomes

Planned aspects		Emerging aspects
Challenge	Working on a real-life case<br>Solving a business problem<br>Working with client/stakeholders outside university<br>Working with a real company	Motivated and curious fellow students that want to have impact<br>Intrinsic motivation to perform<br>Being thrown in the deep<br>Being approached as professionals

	Connecting with field of work<br>Opportunity to contribute to a sustainability solution<br>No choice in challenge	Getting help from teammates<br>Struggles in team<br>The atmosphere in the group<br>Shared responsibility<br>Release control
Team & Mentoring	Working in a team<br>Interdisciplinary team<br>Different personalities in team<br>No choice in teammates<br>Intensive mentoring<br>Feedback from mentor on personal development<br>Feedback from mentor on team process<br>Close relationship with mentor<br>Intensive collaboration in small teams<br>Team meetings<br>Team building exercises<br>Peer feedback	Time pressure<br>Freedom<br>Independence<br>Critical reflection on process<br>Critical reflection on yourself<br>Awareness of learning<br>Learning from each other in team<br>Being accountable for your own development<br>Respect for each other in team<br>Being prepared for the real world in which you will get harsh feedback<br>Having a moderator during team meetings to create the open and respectful atmosphere
Program General	International environment<br>Weekly reflection reports<br>Teachers / mentors<br>Creative exercises during workshops<br>Playfulness of workshop exercises<br>Activation in workshops<br>Giving input in workshops<br>Design Thinking workshops<br>Check-ins & Check-outs<br>Dragon's Den: feedback from experts outside university<br>Critical and constructive feedback<br>Feedback from different angles<br>Taekwondo workshop<br>College tours: inspiring speakers / role models<br>Prototype party<br>Discussions after presentations<br>Selection interviews	Being forced outside comfort zone / frame<br>By working in a team, you learn how you behave in a team<br>Creative tasks stimulated group process in understanding each other<br>Think different through creative games in workshops<br>The playfulness triggers to be creative<br>Learning in a fun way<br>The experience of being open to each other's ideas<br>Thinking outside the box<br>Prepare for workshops<br>Follow up on feedback by improving prototype<br>Opportunities to speak up and take the stage<br>Safe environment<br>Encouraging to be open and vulnerable<br>Dealing with feedback and failure
Design Thinking	Apply Design Thinking to project<br>Apply creative techniques and skills immediately<br>Learning skills for problem-solving in a practical way<br>Design Thinking Toolkit<br>Going through the stages of design thinking<br>Brainstorming<br>Making a prototype<br>Visualizing<br>Working in iterations	

7 THEMATIC ANALYSIS

Elaborated version of description of themes

Here we present the full thematic analysis, including a more elaborated description of the themes and the intermediate steps we took to reach the results presented in the main article.

Theme 1: The value of interdisciplinarity

According to the interviews, vlogs and surveys the students that took part in the Da Vinci Project acknowledge the value of interdisciplinarity. Many students say that getting 'outside their bubble' was the reason why they registered. Many see the experience of collaborating with people from different scientific backgrounds and different approaches to problem-solving as one of their key takeaways. Some emphasize developing better understanding and being more open towards other people's perspectives and opinions.

> 'It's valuable because it makes me feel I should be more inclusive to other's opinions, and I shouldn't be stubborn to my opinion. (...) I learned that I can be wrong.'
>
> 'You don't have to be right or agree, understanding can be enough.'
>
> 'And I think this overall experience has made me more open to different opinions, more aware of the different levels and types of issues that are present when you're trying to implement change.'

Others stress how much they learned from their peers and how it expanded their personal scope of thinking.

> 'I've really, really grown to value how much different perspectives and different backgrounds enhance my learning.'
>
> 'If you really have to work together or find creative solutions or approaches, then you just notice that people really work and think differently and that you can learn a lot from each other there.'

Some students emphasize the value of an interdisciplinary team in problem solving.

> 'In the end I think there are a lot of people trying to solve the same problem. And if everyone always does a little bit in their own corner, then... You could learn a lot from each other, even from studies where you might initially think I don't have to take that into account at all.'

> ‘So, all of these different perspectives really highlighted how we can add to each other's points of view and really collaborate to make a great project and see things that others might have missed and just build up on each other's ideas.’

Learning how your own discipline can contribute to problem-solving opposed to other disciplines and how different disciplines view different aspects of an issue and therefore an interdisciplinary team can tackle the whole together are aspects that are mentioned several times. Some not only mention they learned how to use everyone’s discipline to deliver good results, for example to generate ideas they never could have thought of individually, but also how to integrate and synthesize different perspectives and develop integrative solutions.

> ‘I am a fairly impatient person, so you have to be patient to actually explain concepts to people or to ask how a concept works. And for me it was a good learning experience to subsequently find out that together you can achieve more than just the sum of your knowledge.’

> ‘It's not just that you have a bunch of people in a room and that each have something new to contribute and then you just sum those up**.** It's very much that by each person contributing, they actively change the way that other people contribute. And I think in a positive way because it's the reflective process.’

On the question why they think these learnings are important they give several different answers. Complex issues around sustainability must be tackled interdisciplinary. In their future (scientific) work-life, they will need to collaborate with people with different backgrounds. To have an open attitude towards others and be able to really listen to people is something that you can use in many professional contexts.

> ‘In the end, it's important to explore and expose students to new philosophies to take on fleeting problems. Creating an environment that results in a clash of ideas can create an amalgamation of ideas from different backgrounds, which not only follows, allows the team to progress, but also individual development to occur.’

Theme 2: New ways of thinking

Working in an interdisciplinary team and applying design thinking changes thinking habits, it broadens the student’s perspectives and mindset. Most of the students say they learned news way of thinking.

> ‘I learned new ways of thinking and new ways of looking at situations. The workshops changed my perspective and made me look further than the things I already knew.’

> ‘When I think about Da Vinci, I think about kind of a metacognition perspective in the sense of that Da Vinci is a way to learn how to learn, if that makes sense. It's a way to kind of go in and deconstruct your conceptions of what it means to learn and to think of a new process and to innovate.’

Many students refer to thinking outside the box, creative thinking, divergent thinking, thinking in possibilities (or options) instead of imitations and the ability to escape from obstinate thinking patterns.

> ‘I think I've just, you know, started looking at the world differently. Before that when I was on the bike, I put my earphones in and then I just didn't think about anything. And then I just looked around and I thought. Oh, this can be different, or that can be different.’

> ‘I do think that way of thinking. So that you, well, just try very hard to keep an open mind and think creatively and not get stuck in the thinking pattern you've come up with in the beginning of the process. Just say that. And do that with a group. I found that very valuable.’

Several students mention they learned new ways of approaching (sustainability) problems. Some refer particularly to framing, to the ability to view problems from multiple perspectives as one of their major learnings.

> ‘That is looking through other lenses. And coincidentally with our case, which really played out in a completely different context, but also with my teammates who also looked at the problems very differently. Yes, I think most of it actually.’

In line with this are the learnings students mention about empathy and the awareness of biases. Some students mention the importance to really understand the people you design for as a major learning and considering all stakeholders while developing solutions.

> ‘That you really have to look very closely from all stakeholders or all those involved in such a project at what they want exactly or what they especially don't want. And that you start looking at it, okay, from that perspective, how can you solve such a problem?’

> ‘And it also showed me that I hadn't properly grasped what human centered approach and design thinking actually means. Only going to Rotterdam and trying out the sampling kit in the rain and experiencing the pains that citizen scientists might go through. I really showed me what this means, and it helped me getting rid of, or at least being aware of my own biases a bit better.’

A last new way of thinking which is mentioned as a major learning by some is critical thinking.

> ‘Critical thinking, not in the pure academic sense, but to challenge each other in a constructive way and really think why we would go in a certain direction.’

Theme 3: Authentic challenges

The authentic context of the challenges is one of the most important aspects of the Da Vinci Project. This ensures major learnings. Working with a real stakeholder and on a real-life challenge made the Da Vinci Project a meaningful learning experience according to the students.

> ‘I learned a lot from everyone, but especially I learned a lot from working with a company.’

> ‘Working together with a real partner on a real problem in the real-life world was just really eye opening to me because there's so much more than just solving the problem with a simple solution. Because you're working with people, you need to build relationships, you need to build mutual trust, and you're also going to encounter setbacks where you can learn from and where you can grow from.’

Students mention a variety of insights they gained about the professional field of work, for example in understanding business cases, all kinds of technical aspects of specific sustainability issues, technicalities about certain industries, and about the importance of professional communication. Several students say that they now have better understanding of what they can do after they graduate, because they now can picture work-life outside university better.

> ‘We learned the lesson of scale that it's important to consider actors and stakeholders in all levels and not just on the top, where we ideally want to create a project for.’

> ‘Going to a company and talking with them and really seeing how a company like that functions and like the considerations that a big company makes.’

Also, students mention that they learned that sustainability issues are much more complex than they initially thought.

> 'What I really took from it was that even if you are given the task of designing or making something more sustainable as simple as a lab coat. It's not simple. It's super complicated and messy. And there are reasons why products in our world are maybe not yet sustainable. That was really a great learning I had afterwards.'

> 'In general, I think I changed my radical perspective of: "We need to do everything sustainable now," to like more "No, there are reasons why it's hard to work on that and why it takes such a long time." I guess I'm also more relaxed about sustainability or see more that it is complex. And that's why I guess people and politicians are not working as fast as some of us would maybe want them to.'

Some students express how much working with a real client and a real-life challenge enhanced their motivation and engagement, which in turn enhanced their learnings because they wanted to perform better.

> 'And I put so much time and energy into Da Vinci, so much more than I expected. Just because (...) the vibe of working with a client and what it was like at Da Vinci was just very exciting, so to speak.'

> 'The Da Vinci Project really challenged you. You have that stakeholder, who has a real interest in this subject, and it also motivated me a lot more (...). We soon have that presentation, which is not only for our supervisors, but also for that stakeholder who want to really see something.'

In some interviews students talk about their experience of being approached as professionals and being taken seriously which particularly motivated them. Other students say that it was the fact that they could contribute to a real sustainability issue that was motivating.

Theme 4: Design thinking, creativity, and creative confidence

Developing creativity and gaining creative confidence is an important outcome of going through the process of challenge-based learning and applying design thinking. A lot of students mention creativity as one of their major learnings. Several students say they became more creative, or they gained awareness of their creativity. Sometimes they refer to

creative thinking, the ability to think outside the box or generate creative ideas, as highlighted in the previous section. Sometimes they refer to learning to apply design thinking tools and creative techniques and skills such as visualization and building prototypes.

> 'So before joining Da Vinci, perhaps I didn't see myself as that much of a creative person. But now I know that it's going to really develop yourself into thinking more creatively, and I will definitely be thinking more creatively and seeking out more creative ideas and projects in the future.'

> 'I really felt like I lacked this creativity skill. But actually, during the project, even at the kickoff day already, I found out that I didn't miss it. I just never use it because I was scared and I never tried and I felt ashamed. But these creative thinking workshops really helped me to put that aside and to start creative thinking.'

> 'However, it was hard for me to be very creative and be more broad in my ideas. It was easier to just focus on the data and based on my findings on that and I really had to be more open. And I think this experience is very valuable for the future as it's my goal to go into a consultancy. And I think design thinking is very, very important in this sector, right? Because you have to be fast, you have to think outside the box. You have to be very creative in finding solutions that aren't already there. So in that sense is it really helped me to think about how I should be more broad and more open in a way and not just focus on the numbers, which I usually do.'

> 'I learned the benefits from visualizing not only to explain some things better to others, but also to see for yourself what is actually wrong in the idea that you had in your head.'

Structured ideation is also mentioned as a major learning. But design thinking does not only provide structure to specific phases in the problem-solving process like ideation, by applying design thinking as a methodology students gained the ability to structure the whole process of complex problem-solving.

> 'Of course, you have given all those design workshops and in that chaotic process there were certain handles in that respect. And how best to tackle that. And I took that with me as well. I have learned a lot from that. How you can approach such a process so that it is still somewhat structured and directional.'

> 'The process we were in, so to speak. All that. Yeah, that circle just got me a lot. And also that we were going to do it twice, so to speak. Yes, I just really liked that too. Then it doesn't fall, I think..., then it doesn't have to be right the first time.'

The methodology prescribes a deep understanding of the problem and of the people you design for to develop better solutions. Some students mention the insight that exploring the problem or finding the problem behind it before developing solutions is essential as an important learning.

> 'That is empathizing and patience, because I used to try to jump into solving a problem right away and then being frustrated when it didn't work. And for example, in one of our first meetings, I remember saying that our prototype was probably going to be a modification of the sampling kit. In the end, it ended up being an app. So, I think it was very nice and very important to take the time to get accustomed with the problem properly, delving into what is actually asked.'

> 'And you keep going back and back and you might be able to find a more root issue that requires less resources to address, or it might redirect the way that you approach the whole outcome in the end.'

> 'I think actually that's the most valuable thing I learned from that, because for the last two years, I'm working as a product owner. While I'm doing the product design, I realize how important it is to understand the end user, because if they don't want that technology or they don't want that function, then the function we think is cool is useless.'

This design thinking process is iterative, and some students also address that as a learning outcome. They learned iteration is inherent to working on challenges with stakeholders. You propose something, you receive feedback, you adapt it, and this is repeated until the final deadline.

> 'But we also really had to trust the process, like start again when something is wrong, accept when our ideas were not right and try to fix them. And that way we really came to a really good prototype in the end.'

> 'Even if something went wrong, you could simply consult with that stakeholder and they could say: "Yes, I had a different picture," or "It was not quite what I expected," and then you would have much more re-iteration and then you could go in a completely different direction.'

> 'Yes, just let go of that perfectionism a bit and think: "Okay, this is version one. We'll work through to version twenty-five and finally it's there." And then it's okay. That's a mindset that I think is very important.'

> 'I think the Da Vinci Project really kickstarted me into that... Yes, just see things more as a process rather than one... Instead of a line from A to B, but that it really is a line from A and then twenty thousand times in the other direction. And then you finally get there.'

Another characteristic of the design thinking process exploring multiple solutions by prototyping. There's not just one right solution and you can only find out what solutions might work best if you explore multiple possibilities. This insight is mentioned a few times as a learning outcome by the students. While prototyping, students encounter successes and failures, which is part of the deal, because you can't know upfront whether the envisioned solutions work. When students accept this, there is much more room for learning. The iterative process then stimulates learning outcomes on the level of mindset and behavior such as more resilience, more perseverance, more comfortable with uncertainty and less fear of failure.

> 'And I'm also more resilient to challenges because I will never give up. Like Dyson and his 5000 prototype attempts.'

> 'The other learning goal I had was to become confident in uncertainty. And I call this the biggest learning goal at the beginning of the project. And I can actually still see why because I feel like I've made a lot of progress with this learning goal. And I think I can say that I'm most grateful for having learned this during The DaVinci Project.'

> 'So, for me it was quite obvious because my grades have gotten a little higher and I feel like that was partially thanks to learning creative confidence and just this way of thinking.'

Having the courage to use creativity, to use creative tools and skills, to create, prototype, and test and the ability to learn from it, can be gathered under the term creative confidence. Several students mention growth of confidence in the interviews and vlogs. This cannot be separated from feedback and reflection. It is not only the design thinking process and working with a challenge that stimulated this growth, but the focus on reflection and feedback as well.

> 'The Thursdays meetings and how to deal with feedback and failure definitely had a big influence in achieving it. They really helped me become more confident.

> ‘This entire process definitely made me more confident. And during the prototype party, I was really excited to talk to people, and I didn't feel as awkward as I used to.’

> ‘I learned to never give up and embrace things that are beyond my comfort zone. And I'm very proud of my own personal learning curve and of our accomplishments of with the Green Engine as a group.’

Theme 5: Focus on feedback and reflection

As we have seen in the previous paragraph the project is characterized by continuous learning through reflection and feedback. Students reflect and receive feedback on three aspects: personal development, team performance and project results.

One learning outcome of this process students regularly mention in their interviews and vlogs is the ability to deal with critical feedback and the insight that feedback is an opportunity to learn and improve. Opposed to courses in which assessments and feedback only take place at the end (if feedback is given at all), in an iterative learning journey, students can reflect on and adapt to the feedback continuously. As an effect, students focus more on their learning process instead of the results and students tend to grow more confidence.

> ‘So one obvious example for that is feedback. I think before this project I really could only see feedback in a negative way, but learning from things like the speakers of the college tours, but also the workshops and especially the Dragon's Den, I found out that feedback and uncertainty are something that can be twisted into something super positive because you can only learn from it, revise your actions and eventually get to a better end station.’

> ‘I also learned to take rigorous decisions that scared me in the beginning. [...] I way more trust myself that I can do things.’

Students learned about themselves in this project, about their abilities, about their strengths and weaknesses, and how they function in a team. Through weekly reflection reports and personal feedback from their mentor, the students become more aware of what they learn. At the beginning students set their personal learning objectives and during the project they have their individual learning experience. Guided by the mentor they develop the ability to steer their personal development and they soon take over complete ownership of their personal learning journey. It makes them more active and engaged learners,

because they see their progression and can pursue the learning objectives they value personally. Therefore, every student has different learning outcomes. Introvert students learned to be more assertive, others mention how they learned to let go of being in control. Some students address that they took the opportunity to try out a leader role. They developed leadership skills or discovered whether a leadership role fits them or not.

> 'Yeah, so I used to be quite shy, and I think that was my main problem going in, and that is something that I made huge progress on.'

> 'So, I took a position of a more visible action and visible voice, for example, in communicating my thoughts or so on. I also found that in the later weeks of the program, I found myself voluntarily putting myself in the spotlight, whether in the Ocean Cleanup project or just asking more questions. [...] Lastly, becoming one of the hosts of the prototype party was an incredibly daunting task for me to stand in front of the audience, but I managed to pull it off, receiving multiple compliments from mentors and colleagues.'

> 'Normally I want to like take the biggest part of a project. So, then I know for a fact that I have to control about that and can make something good of that. [...] Especially the trusting part was kind of hard because sometimes I just wanted to check up on their work and see if I could do something. But then I had to stop myself, like focus on your own part and trust them doing their job, which I did.'

Critical reflection is indicated by many students as a meaningful take away. A few students address self-reflection as a learning outcome, others emphasize learning to critically reflect on the team process in the weekly reflections and team meetings.

> 'The Da Vinci Project, we had to write a reflection on paper every week. And this might be one of my goals that has been developed the most, although it was not one of my goals, but unintentionally, I did learn about a lot about this. I was struggling with my reflections in the beginning because I was only writing down facts and summaries about what we had done. And halfway through there was a turning point where I realized I was not doing it correctly and that I should focus on my own personal learning goals. So, what do I want to get out of The Da Vinci Project? What do I want to learn?'

Through reflection and feedback during team meetings students learned how to improve their communication and collaboration for a better team performance. Instead of conflict-

avoiding behavior students learned to address frustrations and conflicts in team meetings facilitated by the mentor. Some mention the insight that addressing frustrations in team meetings improved team performance was one of their major learnings.

> ‘I wasn't expecting to face that many internal challenges which we've obviously had. And yeah, I think that's interesting because you don't learn…, because I’ve really learned to deal with stuff like that.’

> ‘Another thing I really learned during this project is that everyone struggles with group dynamics once in a while. It's not only me who feels frustrations, and I never kind of realized that the best way to solve this is to actually report those frustrations back to my group.’

> ‘So, actually sharing his frustrations improved our group dynamics. So that was a really important learning step for me.’

> ‘We became a lot more capable as a group to face external challenges once we had addressed our internal challenges. So, I think that was very helpful. And even though it wasn't fun, it was definitely maybe quite a steep learning curve.’

Process of thematic analysis

In this section we provide insights in the analysis of interviews, vlogs and surveys on the Miro board by showing the intermediate steps towards the description of findings in the article. First, all labelled data after color coding was transferred to the Miro Board (Figure S5a, S5b, S5d). Then, insights from individual cases were clustered and patterns were discovered (Figure S5c, S5e, S6). Thereafter, the themes were identified (Figure S6, S7). Finally, the relationships between learning activities, aspects mentioned by students, self-perceived learning outcomes, and competencies / IDGs from benchmark models, were graphically mapped (Figure S7, S8).

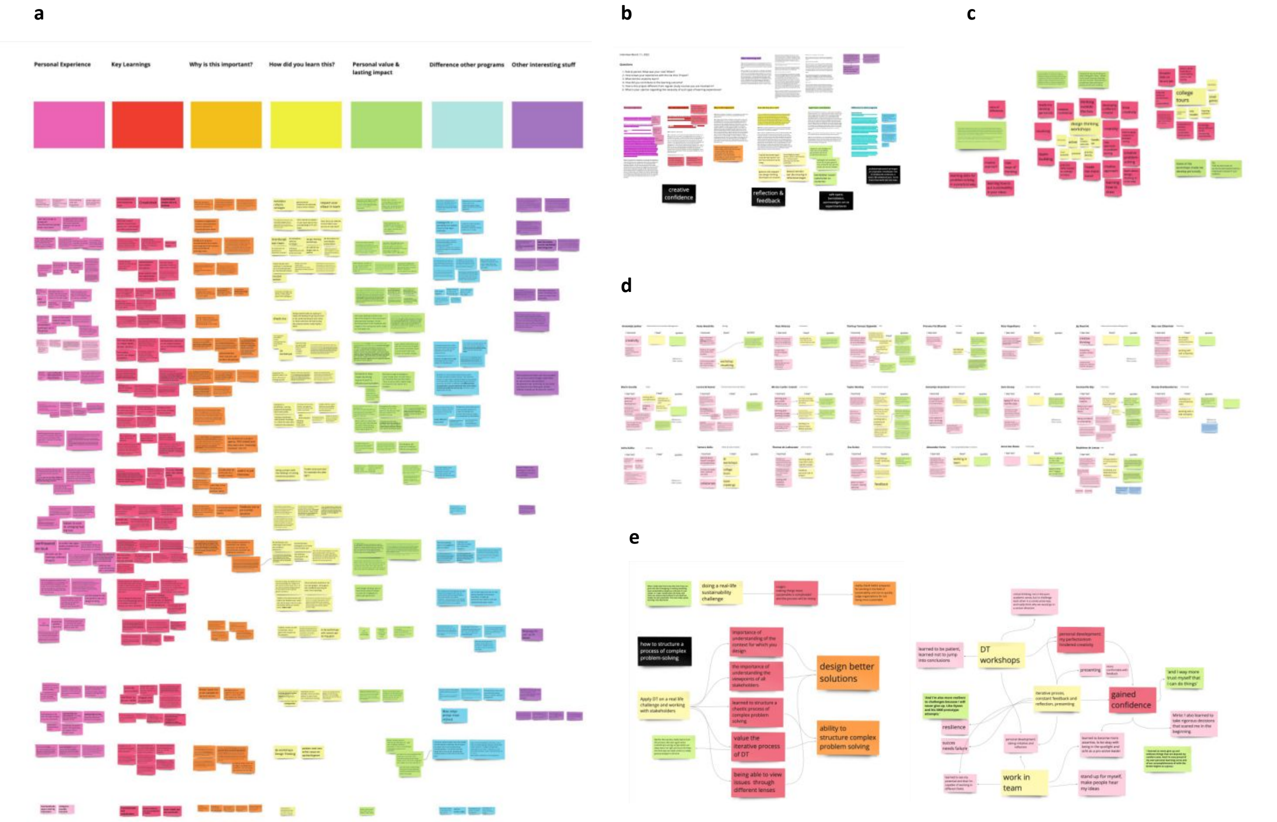

Figure S5. Components of Miro board for analysis. The purpose of this figure is to show the process of analysis, not to read the raw data on the digital sticky notes. Therefore, the raw data are not readable on most of the sticky notes. S5b shows an example of an individual interview with color coding and conversion to colored sticky notes. S5a presents an overview of all coded student interviews. S5d shows an overview of coded selections from the vlogs of one cohort. S5c is an example of an information cluster, analyzing patterns in connections between critical aspects and learning outcomes. S5e consists of three examples of intermediate steps in the thematic analysis.

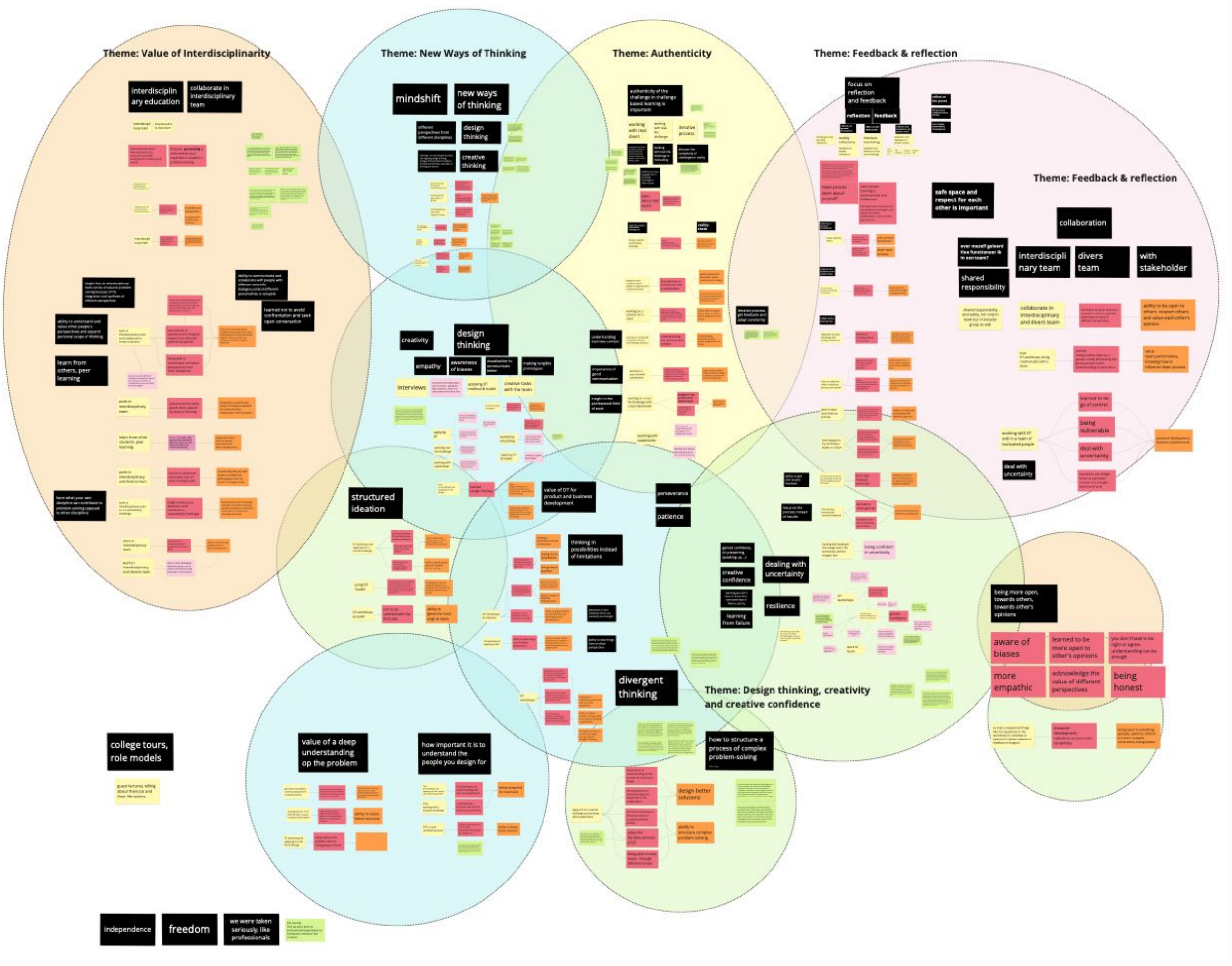

Figure S6. Clusters of insights and theme identification. The colored circles show the five themes: 1. Value of interdisciplinarity; 2. New ways of thinking; 3. Authenticity; 4. Feedback & Reflection; 5. Design thinking, creativity, creative confidence. The purpose of this figure is to show the process, not to read the raw data on the digital sticky notes.

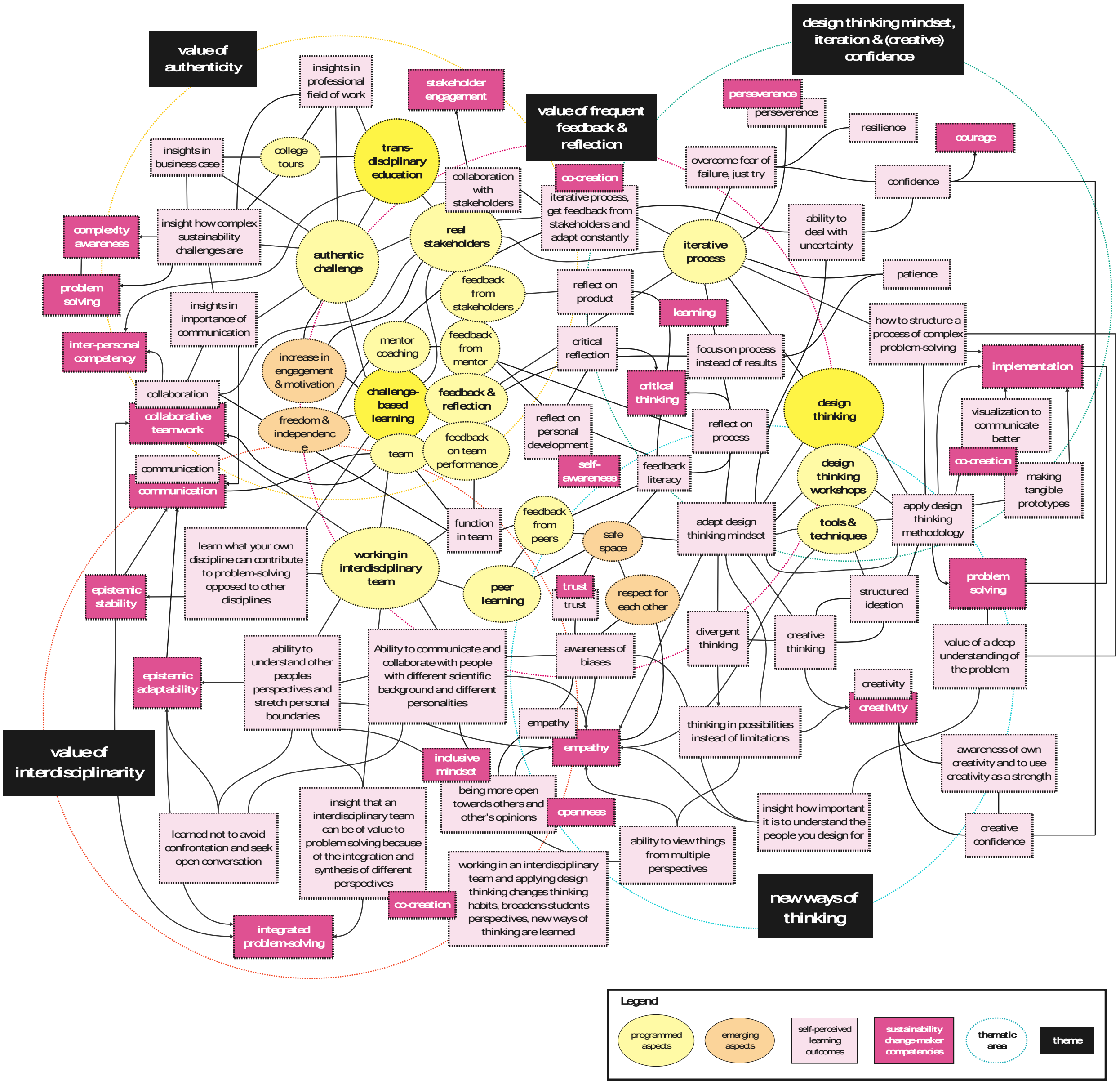

Figure S7. Thematic map of the connections between program aspects and self-perceived learning outcomes. The figure represents the final stage of the thematic analysis in which the connections between the program aspects and the learning outcomes are graphically mapped on a Miro board. The sustainability change-maker competencies (dark pink) are interpretations of the self-perceived learning outcomes mentioned by the students (light pink). The themes are defined by connecting and clustering relationships students mentioned between program aspects and learning outcomes. The purpose of this figure is to show the process of analysis, not to read the raw data on the digital sticky notes.

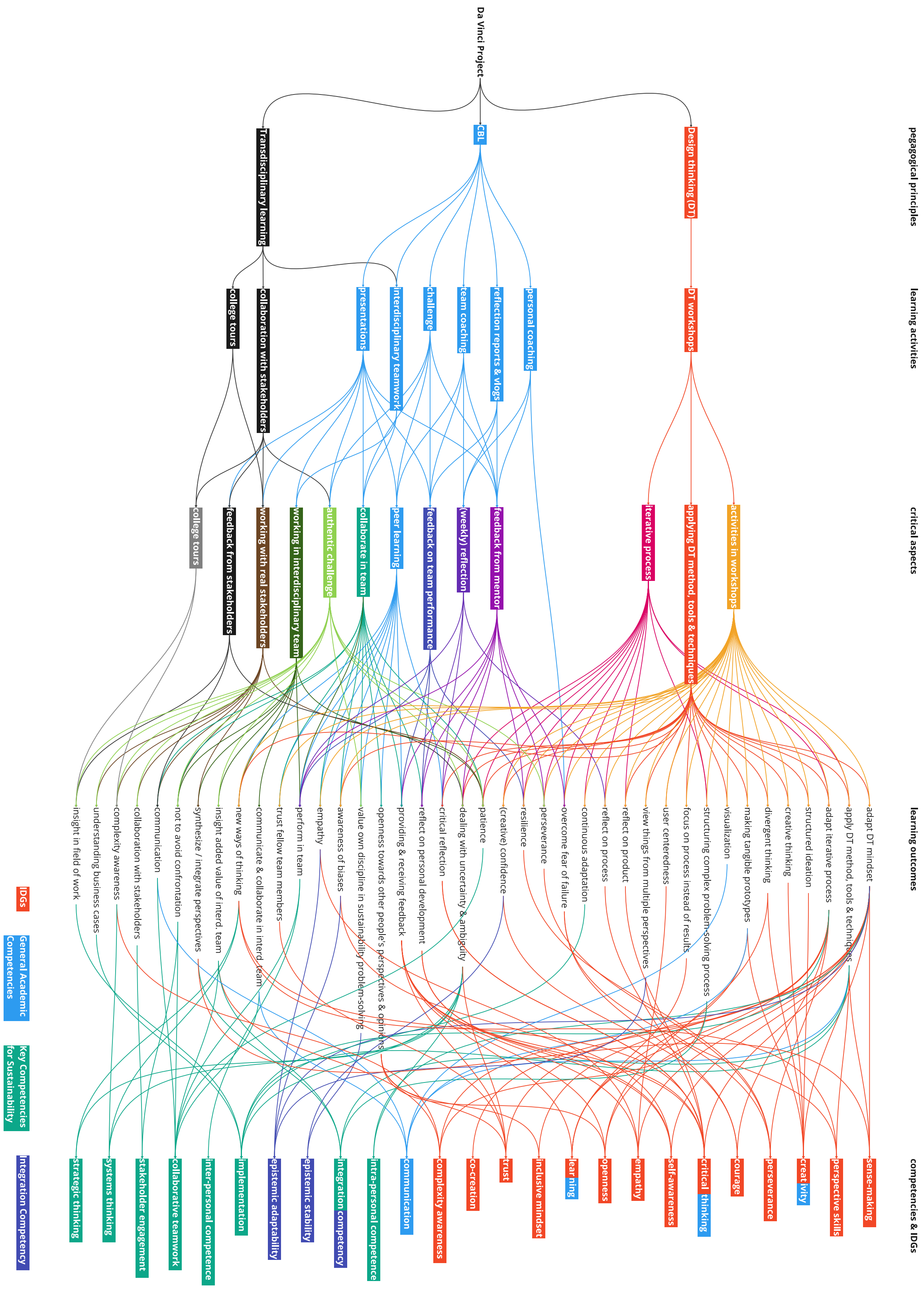

Figure S8. Analysis of relationships between learning activities, aspects mentioned by students, self-perceived learning outcomes, and competencies / IDGs from benchmark models, created on Miro Board.

8 ELABORATED VERSION OF LIMITATIONS

Though a large data set has been collected for this research and a broad analysis has been done, this study inevitably has its limitations. Below we describe the most important shortcomings.

- The conclusions are drawn from what participants say, which causes inevitable biases. There might be a gap between the self-perceived learning outcomes and what they actually learned, which affects the reliability of the results.
- The interviewer was the same person that facilitated the design thinking workshops, which could cause response and confirmation biases and therefore affect reliability.
- Insights in the relationships between the self-perceived learning outcomes and the responsible aspects are gained, but the data did not allow a statistical investigation of these relationships. We cannot draw conclusions on how many students have achieved which learning outcome caused by what interplay of aspects. A further inquiry into these relationships should be done to better understand the conditional aspects related to the learning outcomes.
- This study cannot draw conclusions to whether the alumni of the Da Vinci Project will be successful sustainability change-makers in the future. Most of the participants from the pilot edition were master still students at the time of the interviews. Some of the graduated students pointed out that they believed the Da Vinci Project had been beneficial for their professional life, but this group only consisted of a few individuals. Additional research must be done to understand whether the learning outcomes have lasting effects after graduation.
- We investigated the influence of program aspects on the learning outcomes, but we did not investigate the effects of individual teacher performance. Therefore, different outcomes could be expected when teachers are being replaced or when this program design would be realized at another university. Further inquiry should be done to better understand the influence of the teacher's performance on the learning outcomes.

- We did not study sustainability change-maker competency development in chemistry curricula. Additional research should be conducted to compare the learning outcomes of regular chemistry graduates with those who additionally participated in change-maker education.
- We did not investigate to what extent students developed competencies for sustainable development. Additional research should be done to gain insights in how ascending levels in sustainability competency development can be supported.
- We did not study the differences between editions. Unfortunately, our research data are not suitable to draw conclusions regarding differences between editions. The groups of students spread over the editions are not homogeneous, the team of mentors changed every edition, and, due to for example COVID, there were many other external factors influencing single editions.
- Teaching staff stated in the interviews that the quality of the prototypes improved when the program was extended from 12 to 20 weeks. However, we did not investigate whether the prolongation was beneficial for the students' competency-development.

Da Vinci Project

**Course Coordinator:**	**Prof dr. ir. Bert Weckhuysen (b.m.weckhuysen@uu.nl)**
**Lecturer:**	**Fieke Sluijs (f.sluijs@uu.nl)**
**Coaches:**	**Prof. dr. Marleen van Rijswick (h.vanrijswick@uu.nl)**
	**Prof. dr. Appy Sluijs (a.sluijs@uu.nl)**
**Tutors:**	**Mariangela Biggiero (m.biggiero@uu.nl)**
	**Hannie van Berlo (j.m.vanberlo-vandenbroek@uu.nl)**

Contents

GENERAL INFORMATION 45

COURSE INTRODUCTION 45

LEARNING OBJECTIVES 45

CLASS TYPES AND ACTIVITIES 45

Attendance requirements 45

SCHEDULE 45

FEEDBACK, EVALUATION AND ASSESSMENT 47

Student Reflection 48

GRADING CRITERIA 49

General Information

EC	10 EC
Level	Undergraduate level, to be taken on top of any Bachelor's program (2nd- and 3-rd year students)
Professors	Bert Weckhuysen, Appy Sluijs, Marleen van Rijswick,
Other experts	Hannie van Berlo, Joëlle Siewe, Fieke Sluijs, Mariangela Biggiero

Course introduction

"Our society needs students to become transdisciplinary connectors who are skilled to collaborate outside their own comfort zone and who are moreover able to create innovative solutions on sustainability with different stakeholders."

The Da Vinci project is an interdisciplinary honours program on sustainability. You will meet people from different backgrounds, look across disciplinary boundaries and collaborate outside your comfort zone. You will work together in teams on real-life sustainability related challenges with the involvement of important stakeholders. In the Da Vinci Project you will apply the method of Design Thinking to unlock creativity and solve problems in an unconventional way.

Learning objectives

At the end of this course students are able to:

- apply Design Thinking methods and tools in a commissioned sustainability challenge,
- create a prototype solution for a commissioned sustainability challenge that attends to the commissioning client's goals and the end users' needs,
- reflect on his or her personal contributions to the end result, professional skills, insights and behaviors towards interdisciplinary problems and their own learning process.

Class types and activities

140 contact hours, including 2 fieldtrips, 16 workshops on Design Thinking, 2 pitch-presentations, several College Tours, a prototype-party and an evaluation.

Attendance requirements

In principle, attendance of the classes and activities is mandatory. Since the program is extracurricular and takes place outside of regular education hours, it may occur that absence is inevitable. During the Prototype Party and the 2 pitch presentations, attendance is strictly required, unless there is an important factor outside of your control (i.e. other exam). In case of absence, please notify your coach, as well as the tutor (Joëlle and/or Mariangela).

Schedule

Week 1	Sun 11 Sep	Kick Off – 14:00-17:30 Hof van Cartesius
	Mon 12 Sep	Team Meetup – Ruppert 111
	Thu 15 Sep	Workshop Design Thinking 1 Introduction – BOL 2.049
Week 2	Mon 19 Sep	Team Meetup – Ruppert 111
	Thu 12 Sep	Workshop Design Thinking 2 Discover – BOL 2.049

Week 3	Mon 26 Sep	Team Meetup – Ruppert 111
	Thu 29 Sep	Workshop Design Thinking 3 Empathy – BOL 2.049
	Fri 30 Sep	Deadline Design Brief
Week 4	Mon 3 Oct	Team Meetup – Ruppert 111
	Thu 6 Oct	College Tour 1 Bert Weckhuysen – BOL 2.049
Week 5	Mon 10 Oct	Team Meetup – Ruppert 111
	Thu 13 Oct	Workshop Design Thinking 4 Define & Framing – BOL 2.049
Week 6	Mon 17 Oct	Team Meetup – Ruppert 111
	Thu 20 Oct	Workshop Design Thinking 5 Ideate – BOL 2.049
Week 7	Mon 24 Oct	Team Meetup – Ruppert 111
	Thu 27 Oct	Deadline: Frame Boards Session – BOL 2.049
Week 8	Mon 31 Oct	Team Meetup – Ruppert 111
	Thu 3 Nov	Workshop Design Thinking 6 Storytelling – BOL 2.049
Week 9	Mon 7 Nov	Team Meetup – Ruppert 005
	Thu 10 Nov	Workshop Design Thinking 7 Prototyping – BOL 2.049
Week 10	Mon 14 Nov	Team Meetup – Ruppert 111
	Thu 17 Nov	College Tour 2 Marjan Minnesma – BOL 2.049
Week 11	Mon 21 Nov	Team Meetup – Ruppert 111
	Thu 24 Nov	Deadline: Dragon’s Den – BOL 2.049
Week 12	Mon 28 Nov	Taekwondo – TBD
	Thu 1 Dec	College Tour 3 – BOL 2.049
Week 13	Mon 5 Dec	Team Meetup – Ruppert 005
	Thu 8 Dec	College Tour 4 Mark de Boer – BOL 2.049
Week 14	Mon 12 Dec	Team Meetup – Ruppert 111
	Thu 15 Dec	Deadline: Expert Meeting – BOL 2.049
Week 15	Mon 19 Dec	Team Meetup
	Thu 22 Dec	College Tour 5 Maarten Hajer – BOL 2.049
Week 16	Christmas Holidays	
Week 17	Christmas Holidays	
Week 18	Mon 9 Jan	Team Meetup – Ruppert 005
	Thu 12 Jan	Workshop Design Thinking 8 Creative Confidence – BOL 2.049
Week 19	Mon 16 Jan	Team Meetup – Ruppert 005
	Thu 19 Jan	College Tour 6 – BOL 2.049
Week 20	Mon 23 Jan	Deadline: Prototype Party – TBD
	Thu 26 Jan	(Optional) Team Meetup
Week 21	Thu 2 Feb	Deadline: Vlog – NA

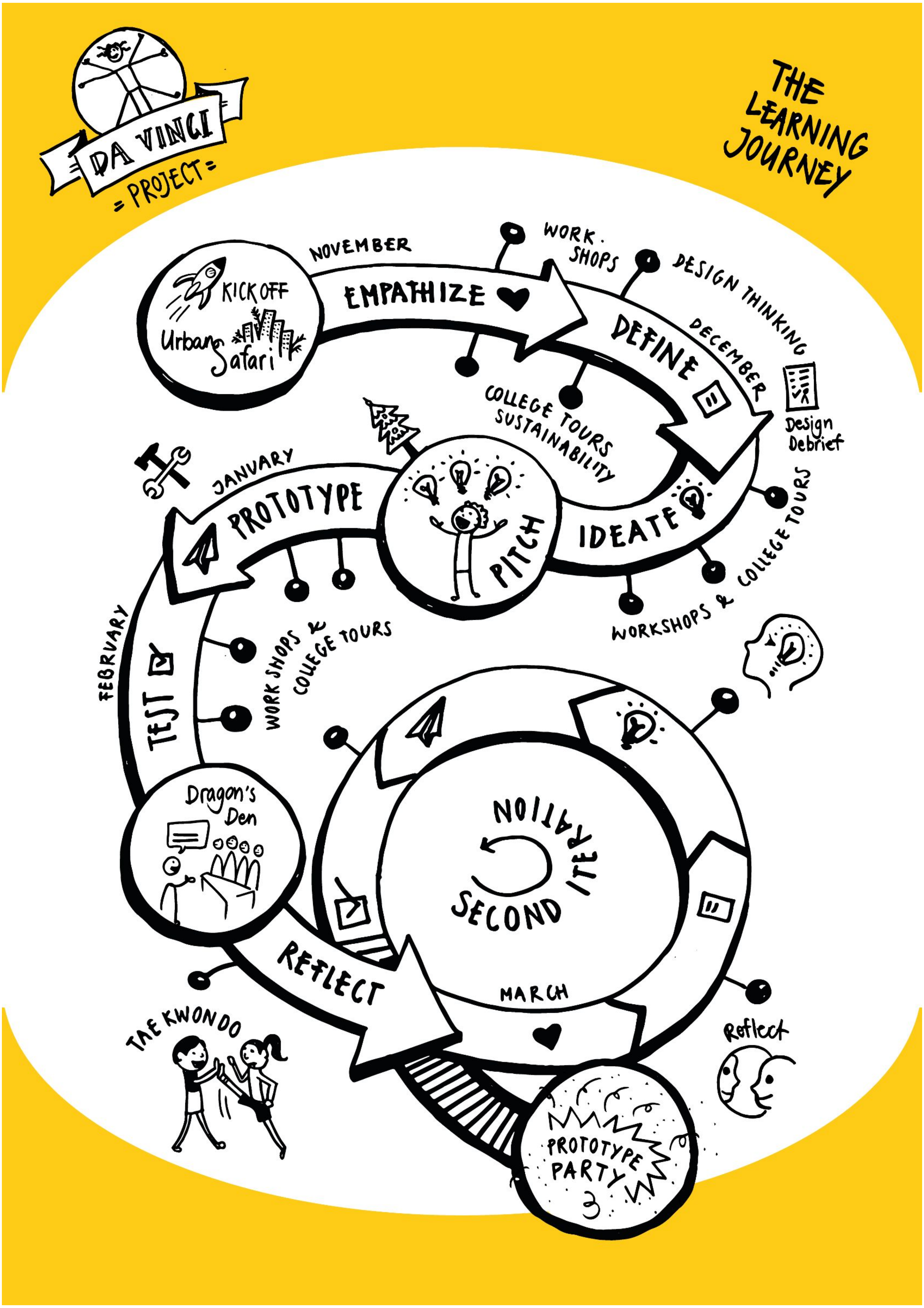

DA VINCI PROJECT
THE LEARNING JOURNEY
KICK OFF
Urban Safari
NOVEMBER
EMPATHIZE
WORK-SHOPS
DESIGN THINKING
DEFINE
DECEMBER
Design Debrief
COLLEGE TOURS SUSTAINABILITY
PITCH
IDEATE
WORKSHOPS & COLLEGE TOURS
JANUARY
PROTOTYPE
FEBRUARY
TEST
WORK SHOPS & COLLEGE TOURS
Dragon's Den
SECOND ITERATION
REFLECT
MARCH
TAEKWONDO
Reflect
PROTOTYPE PARTY

Feedback, evaluation and assessment

- Reflection reports
- Team work
- Reflection vlog

This course is part of the Honours College and the credits earned are extracurricular, meaning they are added on top of the credits of your undergraduate program. As a result, this course is not graded on a numerical scale. Rather, either a Pass (P) or Fail (F) is awarded at the end of the course.

Student reflection

As part of this course, you will be asked to reflect upon your experiences each week. The purpose of reflection is to help you view challenges as part of the normal learning process and to appreciate your own responses to learning. They will give the course instructors a better idea of student engagement with the material.

Each week you will need to submit a one-page (A4) reflection report. At the end of the course, you will submit a five-minute (max.) reflection vlog to your coach. The weekly reflection reports will help you remember and compile some of your struggles and successes with Design Thinking when putting together your vlog.

In your reports, consider reflecting upon the following questions:

- What concept or skill did you learn that week?
- What strategies did you use to solve a problem?
- What did you find confusing or unclear?
- How does your learning process connect to what you already know?
- What would you like more time developing or understanding?
- How are your group dynamics?
- How do you feel when you are working on the course activities?

To get the best out of your reflection exercises, consider drawing or sketching out what you learn. This can also count as your A4!

At the end of the course, the course instructors will review your reflection reports and vlog and give this work a pass/fail mark. In order to pass, we will look at whether:

- The reflection explains the student's thinking about his/her own learning processes;
- The reflection critically analyzes the learning experience; and
- The reflection articulates connections between this learning experience and content from other courses, past learning experiences, and/or future goals.

College Tours

Throughout the program, several guest speakers from fields related to sustainability speak about their experiences in the field and their expertise during a College Tour. You will have the opportunity to ask them questions as well. Each College Tour will be "hosted" by a smaller subgroup. You are expected to lead the College Tour by:

- Providing some general information on the speaker before the College Tour for the other students,

- Welcoming the College Tour speaker and providing the first questions,
- Mediating the questions from the other students.

You are encouraged to be as creative as you want to be for the College Tour you are hosting. For example, you can contact the speaker ahead of time and get their input for some first questions, or host a short gameshow with facts about the speaker and the field they work in.

Frame Boards

The Frame Boards session is your first opportunity to present your ideas for a prototype. You will create three Frame Boards, each detailing a different solution. The Frame Board can be modelled according to the template found in the Design Thinking Toolkit. You will present the Frame Boards to your peers and the teachers, who will provide you with feedback and ask critical questions. Using this input, you can narrow down which prototype you want to create and gain inspiration to expand on your ideas.

Expert Meeting

The Expert Meeting is meant to be a moment of confrontation between your team and experts in the field. The goal is to present your ideas and the development of your project so far, and to get feedback about it. This will not be a formal presentation, and therefore it will take place in the form of Q&A or discussion session with the experts.

Dragon’s Den

The Dragon's Den will take place towards the end of the first iteration of Design Thinking. Just like in the TV show, you will present the first version of your prototype to a panel of experts, called the dragons. How you wish to present the prototype is your choice. So be as creative as you want! The dragons will ask you critical questions about the prototype and provide you with important feedback that you can use to improve your prototype in the second iteration.

Prototype Party

The Prototype Party is the celebratory end of the Da Vinci Project. During the Prototype Party, you will present your final prototype to your stakeholder, but also to your fellow students. Together you will discuss the choices you made for the prototype and why you made them. You may even be able to discuss ways to implement your prototype in real life! The prototype party typically takes place in a fun location outside of the classroom, which will be announced later, and there will be something to drink and some snacks as well to make it truly festive. Apart from the prototype you have made and will bring to the party, we also ask that you prepare a summary of the project you worked on and what your prototype is. We will use this for our own website. Feel free to include photos and/or drawings of your prototype and your team as well!

Grading Criteria

Grades \ Criteria	Not sufficient < 5.4	Sufficient (5.5-6.9)	Good (7-8.4)	Excellent (8.5-10)
1. Empathize & Define<br><br>• User analysis<br>• Context analysis<br>• Design Brief	Based on the briefing, you are able to conduct a problem overview.<br><br>The overview is too superficial. Problem overview is not substantiated with insights from the user and context analysis.<br><br>The design challenge is too broad and is not able to inspire for ideation.	Based on the briefing, you are able to conduct a problem overview and to approach the issue from a human-centered perspective.<br><br>The design brief shows the extensiveness of the issue at hand and explains why the design challenge inspires.	Based on the briefing, you are able to conduct a problem overview and to approach the issue from a human-centered perspective.<br><br>The design brief shows the extensiveness of the issue at hand and explains why the design challenge inspires.<br><br>*+ Primary data were collected that have an added value for the insights.*<br><br>*+ Different tools, such as the Empathy Map, the Context Map and the Challenge Canvas, where used to understand the problem and define the design challenge.*	Based on the briefing, you are able to conduct a problem overview and to approach the issue from a human-centered perspective.<br><br>The design brief shows the extensiveness of the issue at hand and explains why the design challenge inspires.<br><br>Primary data were collected that have an added value for the insights. Different tools were used to understand the problem and define the design challenge.<br><br>*++ Defines multiple challenges to approach the problem from different angles.*<br><br>*++ Shows ability to connect different approaches to the problem, which lead to a distinctive design challenge.*

Grades \ Criteria	Not sufficient < 5.4	Sufficient (5.5-6.9)	Good (7-8.4)	Excellent (8.5-10)
2. Ideate<br><br>• Frame Boards<br>• Pre-pitch	Doesn't show capability for framing insights in an inspiring way.<br><br>Doesn't demonstrate the ability to diverge; sticks to one idea.<br><br>The creative concepts are too similar and/or too obvious.	Demonstrates ability to frame insights in an inspiring way.<br><br>Shows ability to explore a vast amount of ideas and substantiate ideas with insights.	Demonstrates ability to frame insights in an inspiring way.<br><br>Shows ability to explore a vast amount of ideas and substantiate ideas with insights.<br><br>*+ Different creativity techniques for framing and ideation are applied*<br><br>*+ Shows ability to converge (best) ideas into a creative concept.*	Demonstrates ability to frame insights in an inspiring way.<br><br>Shows ability to explore a vast amount of ideas and substantiate ideas with insights.<br>Different creativity techniques for framing and ideation are applied.<br><br>Shows ability to converge (best) ideas into a creative concept.<br><br>*++ Demonstrates ability to develop several distinctive creative frames and/or design several distinctive creative concepts.*
3. Prototype & Test<br><br>• Dragon's Den Pitch<br>• Final Prototype	No (rapid) prototypes were created and prototype(s) weren't adapted after testing.	Shows ability to apply rapid prototyping as a means to assess the value of a concept.<br>Shows capability to adapt a prototype after feedback and testing and explains why it is adapted.	Shows ability to apply rapid prototyping as a means to assess the value of a concept.<br>Shows capability to adapt a prototype after feedback and testing and explains why it is adapted.	Shows ability to apply rapid prototyping as a means to assess the value of a concept.<br>Rapid prototypes are tested before the dragon's den and test results are shared.

			+ Demonstrates improvement of concept and prototype on the base of newly acquired insights.<br><br>*+ Shows ability to reframe the design challenge in second iteration.*	Shows capability to adapt a prototype after testing and explains why it is adapted.<br>Shows ability to reframe the design challenge in second iteration.<br><br>*++ Shows ability to transform the tested design in local context to a concept that suits the global problem at hand.*<br><br>*++ Thinks critically about the context in which the design is used.*

Grades \ Criteria	Not sufficient < 5.4	Sufficient (5.5-6.9)	Good (7-8.4)	Excellent (8.5-10)
4. Reflection<br>• Vlog	Doesn't show ability to reflect on personal performance in the project	Shows ability to reflect on personal skills, insights and behavior in relation to problem solving in an interdisciplinary team.<br><br>Shows ability to contribute to client goals.<br><br>Shows adaptation of feedback.	Shows ability to reflect on personal skills, insights and behavior in relation to problem solving in an interdisciplinary team.<br><br>Shows capability to contribute to client goals.<br><br>Shows adaptation of feedback.<br><br>*+ Shows initiative to enhance the performance of an interdisciplinary team.*<br><br>*+ Shows capability to convert feedback into improvement of deliverables.*	Shows ability to reflect on personal skills, insights and behavior in relation to problem solving in an interdisciplinary team.<br><br>Shows ability to contribute to client goals.<br><br>Shows adaptation of feedback.<br><br>Shows initiative to enhance team performance and assess the value of an interdisciplinary team in problem solving.<br><br>*++ Shows critical assessment of the value of interdisciplinary teams in relation to problem solving.*<br><br>*++ Shows resilience after receiving feedback by continuous improvement of deliverables in*

				favor of learning process.

Week 1

Kickoff meeting (1st period, end of 1st week Sunday afternoon)

Opening event, some design thinking things, informal gathering.

Week 2

First team meetup (Monday)

Introductory presentation about the project. Inform about schedule conflicts. Meet your team. Draft first email to stakeholders, and send it if possible (explain how to email stakeholders, check their email and put the mentor in cc). Tell about weekly reflection reports and preferred method of communication. Also let students fill in preferred tasks.

Workshop 1 – Intro

Introduction to design thinking.

Tasks

Meet with stakeholder asap. **Plan a new meeting immediately** at the end of the first one (e.g. after frame board session). Try to be very involved in the first meetings! For mentors: try to asap discuss if task division is good and **make automated emails** for organization of events.

Week 3

Dreaming about the future: Bert Weckhuysen

Organized by us. Talking about the future of sustainability. Example: discussion between students about different statements. Student should be contacted for content creation (automatic).

Tasks

After stakeholder meeting: students **make the design brief** (from workshop 1). Check it, and help with adjusting if necessary.

Week 4

Workshop 2 – Interviewing

Empathizing with the people you design for, with interview tutorials. Also the **deadline** for the design brief is **this week Friday.**

Tasks

Go interview! Get an idea of what people value to take this into account when designing for them.

Week 5

Workshop 3 – Empathizing and framing

Students make an empathy map, **using data from interviews** that you obtained before. Also the problem statement, design challenge and framing are discussed in this workshop (can also be a separate one, if time allows).

Tasks

Try to frame your challenge, and brainstorm about ideas.

Week 6

College Tour 1

Organized by students. Notify them well ahead of time (1-2 weeks). Mentors and hosts eat together with the speaker beforehand.

Tasks

Work on gathering interview data, framing and brainstorming.

Week 7

Workshop 4 – Ideating

Different exercises are used to let the students ideate on their project. Coming up with the craziest ideas can be very useful. Next step is to converge in preparation for the Frame board Session.

Tasks

Based on what you thought of in the workshop, work out three ideas for the **Frame board Session**.

Week 8

Frame board session

Hosted by us. Bert attends this session. Presentation of appr. 10 minutes followed by 5 min. feedback (time dependent on amount of students) from Bert, mentors and the other students. Make sure people come on time (6 groups = 2 hours). Also discuss the organization prior to the event, e.g. one side of the room to present. Everyone can give input on small notes after presentation, so idea to **provide collection boxes for every team** to take home ideas.

Tasks

It can be useful to discuss the pitched ideas with the stakeholders, so having a meeting in this time would be good. Students can further develop ideas with the input they got.

Week 9

Workshop 5 – Converging and prototyping (2023: Hannie filled in for Fieke)

Pitching ideas, making a storyboard and making a prototyping strategy on canvas. Spaghetti and marshmallow challenge to show importance of early testing.

Tasks

Make 1 prototype for Dragon's Den. Mentors: prepare idea boxes.

Week 10

Dragon's Den

Pitch (not presentation!) + feedback from 3 Dragons in total of 15 minutes. Students host this, but can have a hard time interfering when time is up. Prepare them for this, and step in if necessary. Other groups can give feedback via idea boxes.

Tasks

Use feedback to improve idea.

Week 11

Workshop Taekwondo

Fun physical activity for the students, make sure they come! Also Niels will give an inspiring talk at the end. Students are (unlike their expectations) very positive about this.

Tasks

Continue working on the prototype

Week 12

Workshop 6 – More prototyping (2023: with Fieke this time)

Some videos to show the importance of visualizing your idea in a prototype to convince people and about the way to ask questions (Mom test).

Tasks

Work on the prototype, think about interviewing again to test your design.

Week 13

College Tour 2

Organized by students, same as previous tour.

Tasks

Continue working on the prototype

Week 14

Workshop 7 – Creative confidence

How to deal with failure, build creative confidence.

Tasks

Finishing the prototype.

Week 15

Expert Meeting

Meeting with experts for one-to-one feedback. Starts with short pitch from all groups, then talking to experts. This requires **a lot of organization**, especially with 6 experts. Think about communication, pairing teams to experts, timing, how to transition from group to group, and food and presents. Might be good to take over organization from students, or help them with some kind of template to make sure the event goes well.
Upload all presentations to teams to enable smooth transition on 1 pc.

Tasks

Use feedback to improve design and prepare for Prototype Party

Week 16-17-18

No workshops, only one team meetup in week 15. Weeks 16-17 are Christmas holidays

Week 19

College Tour 3

Tasks

Week 20

College Tour 4

Tasks

Week 21

Prototype party

Tasks

Students make vlogs based on reflection reports and experiences.

Week 22

Optional team meetup and **deadline vlogs on Thursday**

REFERENCES

i. UNESCO. Education for sustainable development goals: learning objectives. Paris: UNESCO, 2017, p10.

ii. Brundiers, K., Barth, M., Cebrián, G., Cohen, M., Diaz, L., Doucette-Remington, S., Drips, W. Habron, G., Harré, N., Jarchow, M., et al. Key competencies in sustainability in higher education - toward an agreed-upon reference framework. *Sustainability Science* **2021**, 16, 13–29; DOI: 10.1007/s11625-020-00838-2.

iii. These definitions are all taken from: Jordan, T., Reams, J., Stalne, K., Greca, S., Henriksson, J. A., Björkman, T., & Dawson, T. Inner Development Goals: Background, method and the IDG framework, 2021.

iv. Felicia, P. Handbook of Research on Improving Learning and Motivation through Educational Games: Multidisciplinary Approaches; IGI Global: Hershey, 2011.

v. Hölzner, H.; & Halberstadt, J. Challenge-based Learning: How to Support the Development of an Entrepreneurial Mindset. In Transforming Entrepreneurship Education. Interdisciplinary Insights on Innovative Methods and Formats; J. Halberstadt, A. Alcorta de Bronstein, J. Greylings, & S. Bisset, Eds.; Springer, 2023, pp 23-36; DOI: 10.1007/978-3-031-11578-3.

vi. Kleijn, R. A. Supporting student and teacher feedback literacy: an instructional model for student feedback processes. Assessment & Evaluation in Higher Education 2023, 48, 186-200; DOI: 10.1080/02602938.2021.1967283.

vii. Design Council UK (sd). Framework for Innovation. https://www.designcouncil.org.uk/our-resources/framework-for-innovation (accessed May 2023).

viii. Baggeroer, D.; Both, T.; Doorley, S.; Ford, C.; Estrada, E.; O'Connor, C.; Witthoft, S. Design Thinking Bootleg 2018. https://dschool.stanford.edu/resources/design-thinking-bootleg (accessed May, 2023).

ix. Schön, D. A. The reflective practitioner. How professionals think in action. Routledge: New York, 1983.

x. Ammon, S. Reflection-in-Action: Donald Schön Reconsidered. The Value of Design Research. 11th European Academy of Design Conference. Boulonge Billancourt, France: Paris Descartes University Institute of Psychology, 2015.

xi. Koh, J.; Chai, C.; Wong, B.; & Hong, H. Design Thinking for Education: Conceptions and Applications in Teaching and Learning. Springer: Singapore, 2015; DOI: 10.1007/978-981-287-444-3_1.

xii. Auernhammer, J., & Roth, B. The origin and evolution of Stanford University's design thinking: From product design to design thinking in innovation management. Journal of Product Innovation Management **2021**, 38, 623-644; DOI: 10.1111/jpim.12594

xiii. Baepler, P., Walker, J.D., Brooks, D.C., Saichaie, K., & Petersen, C.I. A guide to teaching in the active learning classroom: History, research, and practice; Routledge: New York, 2016; DOI: 10.4324/9781003442820.
